# ProQSAR: A modular and reproducible framework for small-data QSAR modeling with fit-and-use models

**DOI:** 10.1186/s13321-026-01175-9

**Published:** 2026-04-22

**Authors:** Tuyet-Minh Phan, Tieu-Long Phan, Phuoc-Chung Van-Nguyen, Lai Hoang Son Le, Van-Thinh To, Tuyen Ngoc Truong, Daniel Merkle, Peter F. Stadler

**Affiliations:** 1 School of Pharmacy, University of Medicine and Pharmacy at Ho Chi Minh City, 41 Dinh Tien Hoang, Saigon Ward, Ho Chi Minh City, 700000 Vietnam; 2 Present Address: Bioinformatics Group, Department of Computer Science & Interdisciplinary Center for Bioinformatics & School for Embedded and Composite Artificial Intelligence (SECAI), Leipzig University, Härtelstraße 16–18, D-04107 Leipzig, Germany; 3 Department of Theoretical Chemistry, University of Vienna, Währingerstraße 17, A-1090 Wien, Austria; 4 Department of Mathematics and Computer Science, University of Southern Denmark, DK-5230 Odense M, Denmark; 5 Department of Chemistry, Texas A&M University, Chemistry Building, 580 Ross Street, College Station, TX 77843 USA; 6 Algorithmic Cheminformatics, Faculty of Technology & Center for Biotechnology (CeBiTec), Bielefeld University, Postfach 10 01 31, D-33501 Bielefeld, Germany; 7 Max Planck Institute for Mathematics in the Sciences, Inselstraße 22, D-04103 Leipzig, Germany; 8 Facultad de Ciencias, Universidad Nacional de Colombia, Bogotá, Colombia; 9 Center for non-coding RNA in Technology and Health, University of Copenhagen, Ridebanevej 9, DK-1870 Frederiksberg, Denmark; 10 Santa Fe Institute, 1399 Hyde Park Rd., Santa Fe, NM 87501 USA

**Keywords:** QSAR, Cheminformatics, Automation, Conformal prediction, Applicability domain, Reproducibility, MoleculeNet

## Abstract

**Background:**

Quantitative structure-activity relationship (QSAR) models are central to computer-aided drug discovery and predictive toxicology, but practical adoption is often impeded by ad-hoc tooling, inconsistent validation protocols, and poor reproducibility.

**Objective:**

We introduce ProQSAR, a modular, reproducible workbench that formalizes end-to-end QSAR development while permitting independent use of each component.

**Methods:**

ProQSAR composes interchangeable modules for standardization, feature generation, splitting (including scaffold- and cluster-aware splits), preprocessing, outlier handling, scaling, feature selection, model training and tuning, statistical comparison, conformal calibration, and applicability-domain assessment. The pipeline can run end-to-end to produce versioned artifact bundles (serialized models) and analyst-oriented reports suitable for deployment and audit.

**Results:**

On representative MoleculeNet benchmarks evaluated under Bemis–Murcko scaffold split, ProQSAR attains state-of-the-art descriptor-based performance: the lowest mean RMSE across the regression suite (ESOL, FreeSolv, Lipophilicity; mean RMSE $0.658\pm 0.11$), including a substantial improvement on FreeSolv (RMSE $0.494$ vs. $0.731$ for a leading graph method). On quantum mechanical benchmarks, ProQSAR demonstrated superior performance on the single-task dataset QM7 and maintained competitive results on the multi-task QM8 dataset. For classification, ProQSAR achieves the top ROC–AUC on ClinTox (91.4%) while remaining competitive across other benchmark (overall classification average $70.4\pm 11.6$). Crucially, all predictions are accompanied by cross-conformal prediction and explicit applicability-domain flags that identify out-of-distribution entries, enabling calibrated and decision support.

**Availability:**

ProQSAR is released on PyPI, Conda, and Docker Hub; all releases embed full provenance (parameters, package versions, checksums) to ensure reproducibility.

**Scientific contribution:**

ProQSAR (i) enforces best-practice, group-aware validation together with formal statistical comparisons across models, (ii) integrates calibrated uncertainty quantification (cross-conformal prediction) and applicability-domain diagnostics for interpretable, risk-aware predictions, and (iii) exposes both a composable developer API and a one-click pipeline that generates deployment-ready artifacts and human-readable reports, demonstrated on representative benchmarks.

## Introduction

Quantitative structure-activity relationship (QSAR) modeling, the computational framework that correlates molecular representations with predicted biological activities or physicochemical properties, has evolved into a fundamental methodology in computer-aided drug design (CADD), predictive toxicology, and environmental risk assessment [1]. By navigating vast chemical space to triage millions of virtual compounds *in silico*, QSAR dramatically compresses the iterative *Design–Make–Test–Analyse* (DMTA) cycle and curtails reliance on resource-intensive *in vitro* cell-based assays or ethically contentious *in vivo* animal models [2, 3]. Since the seminal linear free energy relationship (LFER) analyses of Hansch and Fujita [4], algorithmic evolution has mirrored advances in statistical and machine learning. This progression spans from classical multi-linear regression, through ensemble methods like random forests and kernel-based support vector machines [1], to contemporary graph neural networks that learn salient structural features directly from the molecular graph topology or simplified line-entry notation (SMILES) [5, 6]. Yet, translating these theoretical advances into a robust, predictive, and regulatory-compliant QSAR model remains a complex, multi-stage undertaking. The workflow typically involves comprehensive data curation, generation and selection of relevant molecular descriptors, careful choice of learning algorithms, hyperparameter optimization, rigorous statistical validation, explicit definition of the applicability domain, and systematic quantification of predictive uncertainty. Each of these stages requires substantial cheminformatics expertise and, when conducted through fragmented or *ad hoc* scripting, is highly susceptible to irreproducibility, thereby undermining adherence to the *FAIR (Findable, Accessible, Interoperable, Reusable)* principles that are increasingly mandated for computational chemistry and toxicology [7].

In response to this operational bottleneck, several automated platforms have emerged to streamline the construction of QSAR models [8, 9]. AutoQSAR (2016) [10] is a commercial (proprietary) Schrödinger application that automates descriptor generation, feature selection, model training, and validation within a graphical workbench. The open-source ChemProp catalyzed a shift toward directed message-passing neural networks and transfer learning, offering a turnkey interface for SMILES-based deep learning; however, its explainability is largely limited to substructure-level attributions (e.g., Monte-Carlo-Tree-Search to find rationales), and although it accepts user-supplied descriptors, descriptor-based workflows are not its primary focus [11]. The DeepTox platform (2016)  [12] powerfully demonstrated the utility of multi-task deep learning by dominating the *Tox21 challenge* [13] through automated data normalization, the use of diverse molecular fingerprint inputs, and model ensembling, but its status as a research prototype dependent on large, densely annotated datasets has constrained its broader adoption. More recently, Uni-QSAR (2023) fused 1D, 2D, and 3D molecular representations within an automatic machine learning (AutoML) engine, leveraging large-scale pretraining on unlabeled chemical data to achieve benchmark-leading accuracy; nonetheless, its deployment is gated by a proprietary cloud instance requiring substantial GPU resources [14]. QSARtuna (2024) is a Python/Optuna QSAR workbench that supports classical and graph models and supplies uncertainty quantification and calibration tools; it offers both a CLI and a Python/Jupyter API, but does not automate formal statistical hypothesis testing for model comparisons [15].

Despite substantial methodological advances in QSAR modeling, several persistent shortcomings restrict uptake in pharmaceutical R&D and regulatory settings [16]. Comprehensive internal and external validation is not applied by default across many toolchains, leaving models insufficiently tested against overfitting and information leakage; model behavior in low-data regimes (e.g., sparsely populated congeneric series typical of wet-lab assays where sample size is small) is often unstable and standard pipelines rarely provide data-efficient defaults such as transfer learning, self-supervised pretraining, or conservative feature constraints; and rigorous uncertainty quantification and applicability-domain diagnostics (e.g., calibrated prediction intervals or conformal prediction sets) are commonly implemented as optional add-ons rather than integral, machine-readable outputs [17, 18], which undermines end-user trust and complicates regulatory assessment.

These methodological gaps are exacerbated by practical deployment frictions. Real-world QSAR routinely operates on heterogeneous tabular inputs (assay metadata, experimental conditions, and derived descriptors) that demand deterministic, leakage-free cleaning, imputation, and column-typing before modeling [19, 20], yet end-to-end tools that guarantee such preprocessing and produce inference-ready artifacts are uncommon; likewise, packaging a validated model into a versioned, containerized, *fit-and-use* artifact (one that, once trained, can be used for inference without additional feature transformations, recalibration, or manual post-processing) remains nontrivial and places a heavy engineering burden on practitioners without computer-science expertise. To address these limitations, we present ProQSAR, an open-source Python toolkit accessible as both a Python package for Jupyter Notebook workflows and a command-line interface for automated execution. It integrates auditable validation by default (paired, repeated resampling, and external testing), data-efficient modeling options targeted at small-data scenarios, and built-in, calibrated uncertainty and applicability-domain outputs, while automating deterministic tabular preprocessing and exporting versioned, deployable fit-and-use artifacts to lower the barrier for bench scientists and support reproducible, submission-ready deliverables.

## Methods

### System overview

ProQSAR is implemented as a Python package built around a pipeline-first, strongly-typed architecture that emphasizes deterministic, leakage-free data engineering. The codebase uses PEP 484 type hints and an extensive test suite (unit and integration tests) to promote maintainability and safe refactoring. Core value objects expose small, well-documented APIs to encourage composition and allow independent testing of individual pipeline components. Pipeline wrappers encapsulate every processing step (cleaning, imputation, featurization, scaling, model fitting and calibration) so that stateful transforms are fitted on training data only, their parameters are serialized, and identical transforms are applied unchanged at inference time to prevent information leakage and guarantee reproducibility. ProQSAR is built on top of established cheminformatics tooling (notably RDKit for structure processing and canonicalization [20]) with mainstream machine-learning libraries such as scikit-learn [21], XGBoost [22], CatBoost [23], and Optuna [24]. Detailed dependency versions and system requirements are listed in the Supporting Section S1.

The software supports two complementary modes of use: (1) a *modular library* of composable building blocks for developers (see Sect. “Module architecture”); and (2) an *automated pipeline* that executes a configured modelling workflow from raw SMILES and tabular assay data to validated, deployable predictors with a single command (see Sect. “Automated pipeline and report”). Both modes share the same core components and configuration schema to ensure reproducibility, traceability and ease of extension.

### Module architecture

Let $\mathcal {D}=\{(s_j,y_j)\}_{j=1}^N$ be the dataset, where $y_j\in \mathcal {Y}$ denotes the target. It may be a continuous molecular property, a quantitative or categorical bioactivity measure, a binary label $\{0,1\}$, or a multiclass category. The pipeline is built from small, focused modules:

$$\Phi = \Phi _K \circ \cdots \circ \Phi _1,\qquad \Phi _k: {\mathcal {T}}_k \rightarrow {\mathcal {T}}_{k+1},$$

where modules pass compact, strongly-typed dataclasses $\tau _k\in \mathcal {T}_k$ and expose simple, stable interfaces (see Figure S1). The Data module handles input ingestion and produces a single train/test partition; internal cross-validation splits for model selection are generated and managed by the Evaluation module. When Evaluation constructs the family $\mathcal {S}=\{(\mathcal {T}_{i,r},\mathcal {V}_{i,r})\}$ indexed by fold $i$ and repeat $r$, and flattens it into $n$ blocks $b=1,\dots ,n$, those blocks are consumed by downstream modules during internal validation. Candidate models are collected in $H=\{h_q\}$. For a scalar metric $m$, write $m_{i,r}(h)$ for the score of model $h$ on fold $i$, repeat $r$; the oriented score is $\tilde{m}_{i,r}(h)=s(m)\,m_{i,r}(h)$ (where $s(m)$ orients the metric). The per-block mean for model $h_q$ is

$$\bar{m}_q \;=\; \frac{1}{n}\sum _{b=1}^n \tilde{m}_b(h_q).$$

Finally, $\mathcal {D}^{\mathrm{te}}$ denotes the external held-out test set produced by the Data module for final evaluation.

$\boldsymbol{\Phi 1.}$: Data (Sect. “Data preparation”): read SMILES, standardize/canonicalize, build RDKit molecules, compute descriptors/fingerprints, and produce an external train / test partition $(\mathcal {D}_{\mathrm{tr}},\mathcal {D}_{\mathrm{te}})$.
$\boldsymbol{\Phi 2.}$: Preprocess (Sect. “Preprocessing”): a deterministic pipeline $\mathcal {P}=T_R\circ \cdots \circ T_1$ where each transformer $T_\ell$ is fitted on training indices only. Typical steps: deduplication, imputation, low-variance filtering, outlier handling, encoding and scaling.
$\boldsymbol{\Phi 3.}$: Model (Sect. “Model”): candidate estimators and fitting logic. Under control of the Evaluation module, models in $H=\{h_q\}$ are trained on the training portion of each CV fold; for each $h$ we keep per-fold predictions $\hat{y}_{i,r}(h)$, scores $m_{i,r}(h)$, oriented scores $\tilde{m}_{i,r}(h)$, and block means $\bar{m}_q$. The selected model is $h^\star =\arg \max _{h_q\in H}\bar{m}_q$.
$\boldsymbol{\Phi 4.}$: Evaluation (Sect. “Evaluation”): internal + external validation, statistical comparisons (repeated measures ANOVA / Friedman + post-hoc), applicability-domain checks and calibrated uncertainty (conformal). Outputs include the score matrices $\{m_{i,r}(h)\}$, aggregates $\{\bar{m}_q\}$, paired differences $d_b(p,q)$ and adjusted $p$-values.

All modules $\Phi _k$ are deterministic given their inputs and configuration (including RNG seeds). They declare the input/output dataclass types $\tau _k$ and persist provenance (split indices, seeds, fitted parameters) so every per-fold prediction and later statistical comparison is traceable and reproducible.

#### Data preparation

**Fig. 1:**
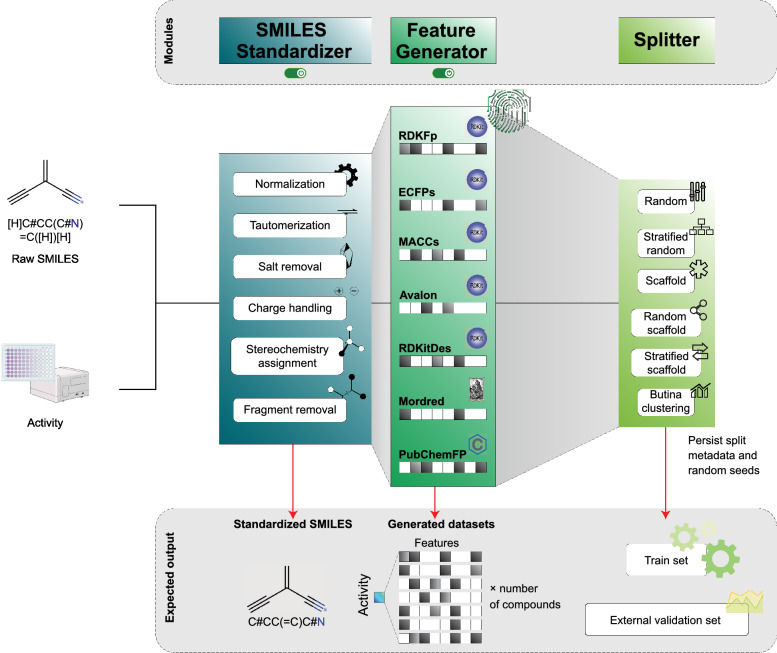
Data preparation workflow: molecular standardization, feature generation, and deterministic partitioning for reproducible modeling

Raw molecular records (SMILES) are normalized and sanitized by Standardizer classes (salt/fragment stripping, tautomer and charge normalization, stereochemistry assignment, canonicalization) to yield deterministic RDKit molecules [19, 20]. The Featurizer then produces compact, interoperable feature blocks using established tooling: circular/hashed fingerprints (e.g., ECFP) [25], substructure keys (e.g., MACCS) [26], and physicochemical descriptor suites [27]. Partitioning is handled by Splitter, which supports random, stratified, Bemis-Murcko scaffold [28], and Butina clustering splits [29]; split metadata and random seeds are persisted to ensure traceability and repeatability. A complete formal specification of the dataset partitioning algorithms is provided in Supporting Section S3, whereas the overall data–preparation workflow is schematically illustrated in Fig. 1.

#### Preprocessing

**Fig. 2:**
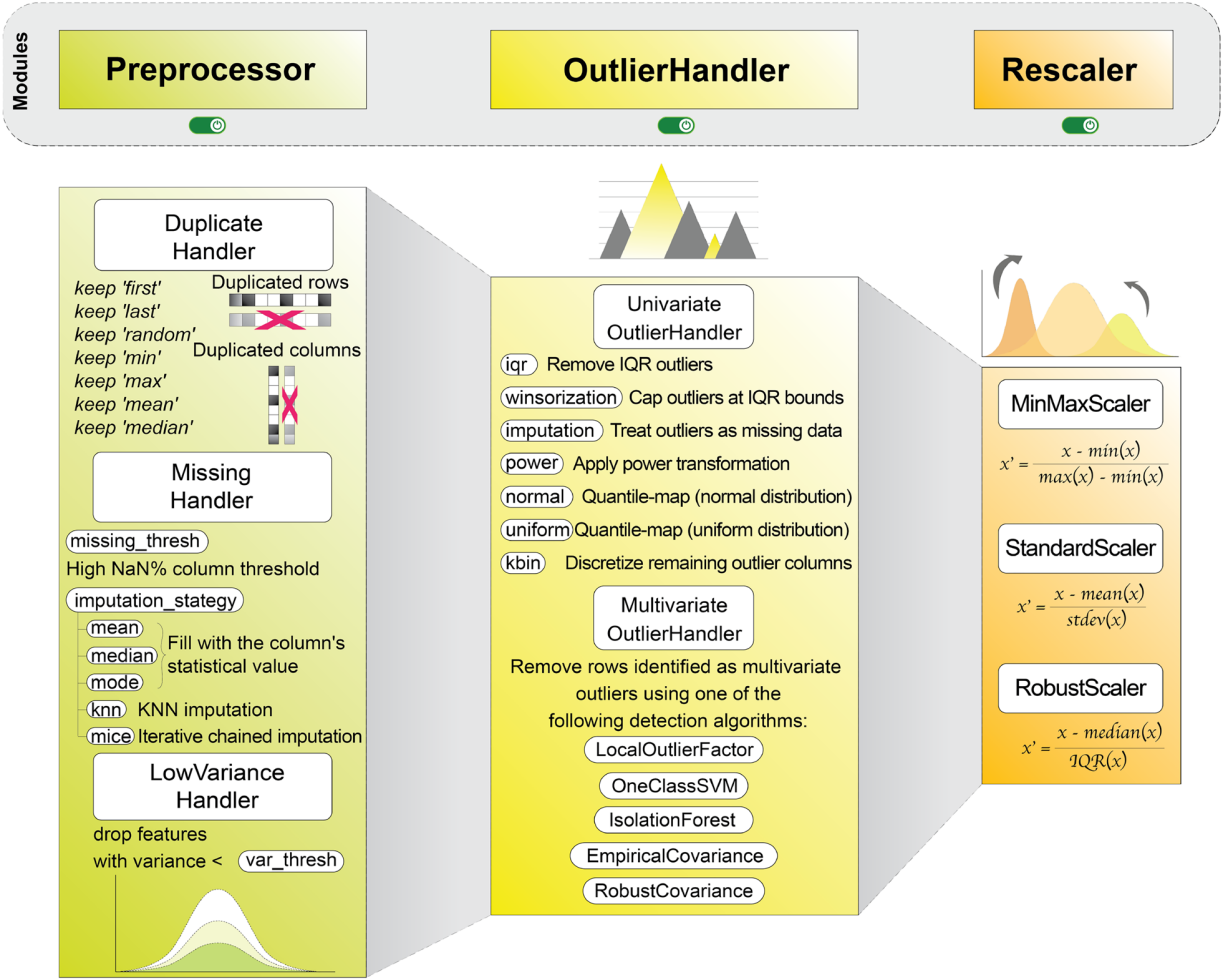
Data preprocessing pipeline: deduplication, missing-value handling, low-variance filtering, outlier mitigation, and scaling

Preprocessing is implemented as a deterministic sequence of stateful transformers

$$ \mathcal {P}=T_R\circ \cdots \circ T_1,\qquad T_r:(\mathbb{R}^{n\times P_{r-1}}\times\mathcal{Z}_{r-1})\to(\mathbb{R}^{n\times P_r}\times\mathcal{Z}_r),$$

where each $T_r$ is fitted exclusively on the training indices to obtain parameters $\widehat{\theta }_r=\mathrm{fit}(T_r,\mathcal {I}_{\mathrm{tr}})$, which are then applied unchanged to validation and test data. This design formally prevents information leakage and ensures reproducible numeric transforms. Because each $T_r$ follows the fit/transform contract, the entire chain can be composed directly within a sklearn.Pipeline, allowing preprocessing to be stacked seamlessly with model estimators and other workflow components. The whole pipeline of this module is illustrated in Fig. 2.

*Deduplication*: DuplicateHandler detects effectively identical feature vectors arising from graph invariance, tautomer/stereochemical ambiguity, hashing collisions, or descriptor degeneracy. Resolution is policy-driven (retain extrema, aggregate by mean/median, or apply consensus) and always preserves identifiers and targets. All decisions are logged in the metadata manifest for audit. See Supporting Section S4.1 for details.

*Missing-value remediation*: MissingHandler is trained on the training fold and supports explicit policies that are selected according to proportion of missing data patterns: column removal when missing value ratio exceeds a configured threshold (default to 40%); univariate imputation (mean/median/mode); and multivariate imputation (KNN, iterative/MICE). Multiple and probabilistic imputation modes are supported where uncertainty propagation is required [30]. All imputation statistics and applied masks are serialized as part of the run artifacts. See Supporting Section S4.2 for details.

*Low-variance filtering and outlier handling*: LowVarianceHandler removes features with zero or near-zero variance according to configurable thresholds (see Supporting Section S4.3). Outlier detection is performed by UnivariateOutliersHandler (IQR fences [31], winsorization [32]) and MultivariateOutliersHandler (Local Outlier Factor (LOF) [33], Isolation Forest [34], One-Class SVM [35], and EllipticEnvelope-based robust/empirical covariance detectors [36]). Post-detection remediation options are as follows. For univariate outliers, implemented options include (a) removing rows that contain IQR-defined outliers; (b) capping extreme values at the IQR limits (winsorization); (c) marking outliers as NaN and imputing them, delegating imputation to the project’s MissingHandler; (d) applying a distributional transform to normal or uniform; and/or (e) discretizing values using KBinHandler [37]. For multivariate outliers, the implemented remediation is row removal only. The selected remediation strategy and its parameters are recorded in the run metadata. See Supporting Section S4.4 for details.

*Scaling and operation order*: Scaling transforms (min-max, standard, robust) are applied after imputation and outlier remediation to avoid amplification of artifacts. See Supporting Section S4.5 for details.

#### Model

Let $\mathcal {S}=\{(\mathcal {T}_{i,r},\mathcal {V}_{i,r})\}_{i=1..k,\;r=1..R}$ denote the shared resampling family (default $k=5,R=5$). For any candidate $c$ (feature strategy or estimator) and scalar metric $m$ (e.g. $R^2$, RMSE) we record the paired per-split scores $\textbf{m}(c)=(m_{i,r}(c))_{(i,r)\in \mathcal {S}}$. All formal hypothesis testing and multiple-comparison procedures invoked below are performed by the Evaluation module (Sect. “Evaluation”).

*Feature selection and model development*: The FeatureSelector and ModelDeveloper components implement a sequential pipeline for automated machine learning under a unified, data-driven evaluation policy. This policy assesses candidates via repeated cross-validation over data splits $\mathcal {S}$, identifying the optimal choice as the one that maximizes the mean of a target performance metric $\textbf{m}$. In the first stage, the FeatureSelector optimizes the feature space by evaluating diverse strategies (e.g., filter methods, embedded methods, and a no-selection baseline). It generates candidate feature subsets $F^{(\ell )}$ and measures their utility using the performance $\textbf{m}\bigl (F^{(\ell )}\bigr )$ of a canonical estimator. In the second stage, the ModelDeveloper selects the best learner within this refined feature space ($F^\star$) by benchmarking a registry of candidate algorithms *h* and computing $\textbf{m}(h)$ on the optimized data. The result of this cascaded optimization is a fully specified predictive model, comprising the optimal feature set $F^\star$ and the best-performing learner, supplemented by comprehensive reports detailing the per-split validation metrics produced at each selection stage.

**Fig. 3:**
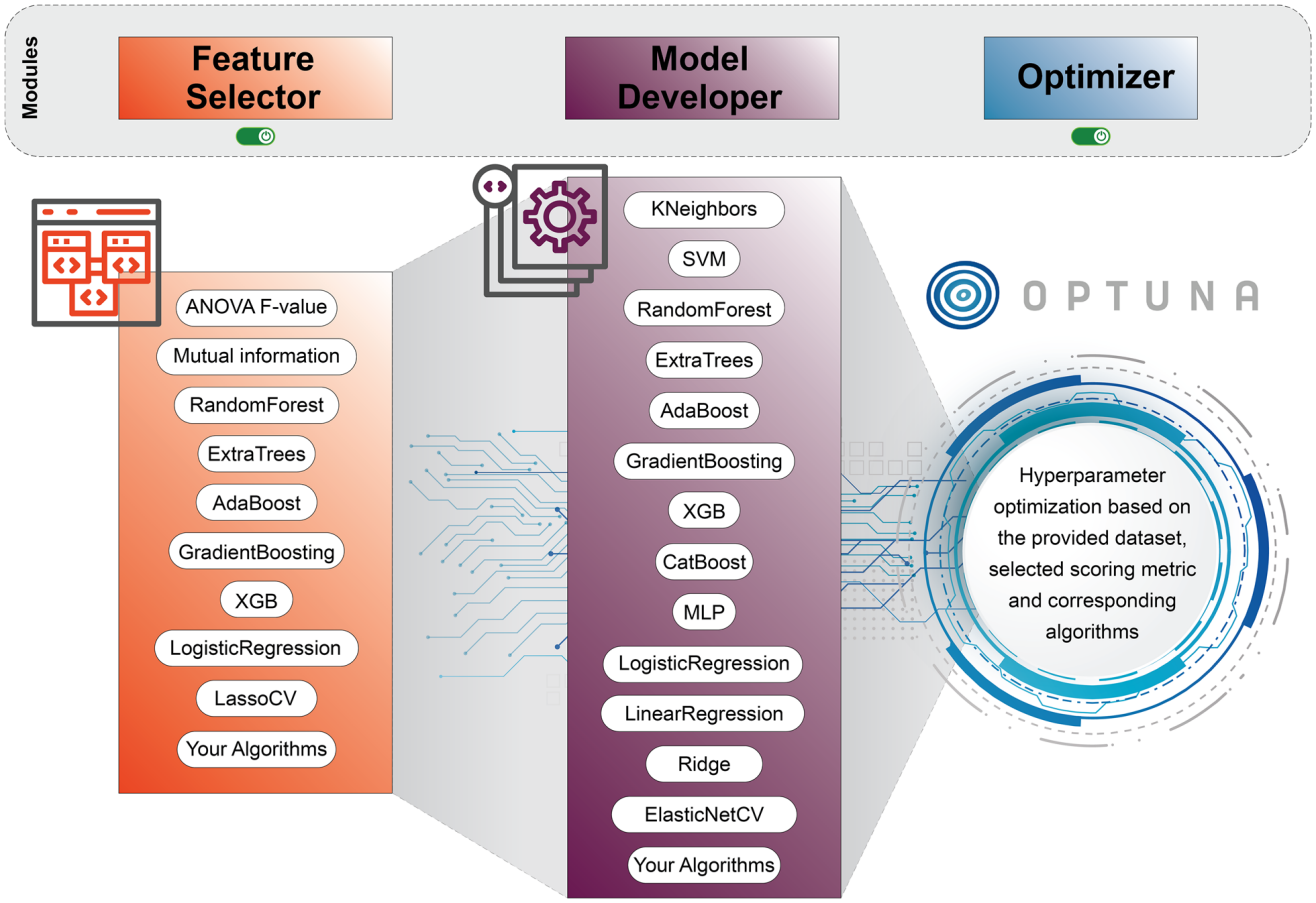
Model development modules: feature selection, estimator benchmarking, and hyperparameter optimization

*Optimization*: Optimizer performs joint hyperparameter and model selection for a list of candidate models by optimizing an objective function $J(\theta )$, defined as the mean score from repeated cross-validation. The search is orchestrated by Optuna, using a Tree-structured Parzen Estimator (TPE) sampler with built-in support for parallelism. To score each trial, every configuration is evaluated within a cross-validation scheme. For flexibility, users can supply custom estimators and define their own parameter search spaces. The output consists of the best-found configuration $\theta ^\star$ (including the chosen model and its parameters), the corresponding cross-validated score, and the persistent Optuna study object, which provides a full history of all trials in Fig. 3.

#### Evaluation

**Fig. 4:**
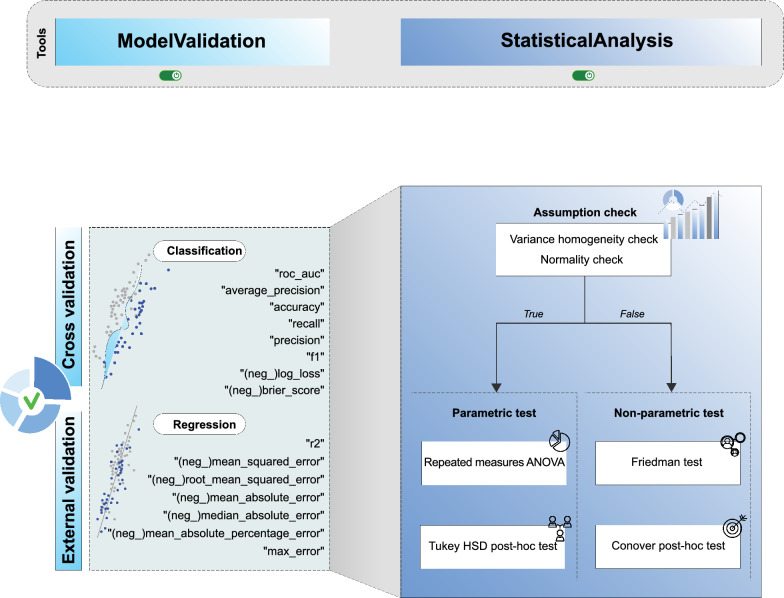
Evaluation workflow: repeated cross-validation over a fixed split family to estimate internal performance and variance; external validation on held-out test set(s) to assess generalizability; and formal statistical comparison using paired tests and multiple-comparison procedures

Evaluation consolidates repeated $k$-fold internal resampling and external testing within a paired, repeated-measures framework. Our approach to statistical comparison is inspired by the work of Jeremy et al. [38]. Let $\mathcal {S}=\{(\mathcal {T}_{i,r},\mathcal {V}_{i,r})\}_{i=1..k,\,r=1..R}$ be the fixed split family (default $k=5$, $R=5$). For any contender $h$ and scalar metric $m$ (regression: $R^2$, RMSE, MAE; classification: ROC–AUC, PR–AUC, accuracy) define the paired per-split score

$$m_{i,r}(h)\;=\;m\!\bigl (h\mid \mathcal {T}_{i,r},\mathcal {V}_{i,r}\bigr ),\qquad (i,r)\in \mathcal {S},$$

and collect the score vectors $\textbf{m}(h)=(m_{i,r}(h))_{(i,r)\in \mathcal {S}}$. Paired comparisons use the same $\mathcal {S}$ across all contenders to eliminate split-induced variance and maximize statistical power. The evaluation process is illustrated in Fig. 4.

*Internal cross-validation* serves to estimate the generalization performance of each contender, thereby facilitating model selection. For a given set of contender models $\mathcal {H}=\{h_1, \dots , h_Q\}$, a repeated $k$-fold cross-validation scheme is employed, using a stratified approach for classification tasks to preserve class proportions across folds [21]. For each model $h\in \mathcal {H}$, the score vector $\textbf{m}(h)$ is systematically computed over the fixed split family $\mathcal {S}$. The resulting per-split scores are aggregated, and may be augmented with summary statistics such as the mean ($\bar{m}$), standard deviation ($\sigma _m$), and median. These empirical score distributions are then visualized to provide a qualitative comparison of model stability and central tendency.

*External validation* provides a final, unbiased estimate of a finalized model’s performance on data held out from all training and selection procedures. The data is partitioned into a single training set $\mathcal {D}_{\mathrm{tr}}$ and an external test set $\mathcal {D}_{\mathrm{te}}$, such that $\mathcal {D}_{\mathrm{tr}} \cap \mathcal {D}_{\mathrm{te}} = \emptyset$. Following this protocol, a final model $h^*$ for each contender $h \in \mathcal {H}$ is fitted using the entire training dataset: $h^* = \text {fit}(h \mid \mathcal {D}_{\rm{tr}})$. Subsequently, the performance of $h^*$ is evaluated once on $\mathcal {D}_{\mathrm{te}}$, yielding a scalar point estimate $m(h^* \mid \mathcal {D}_{\rm{tr}}, \mathcal {D}_{\rm{te}})$ for each metric $m$. These point estimates are complemented by diagnostic visualizations, such as ROC and PR curves for classification or predicted-versus-actual scatter plots for regression, which operate on the predictions made on $\mathcal {D}_{\rm{te}}$ to offer deeper insight into model behavior.

*Assumption checks*: Prior to parametric inference, homogeneity of variances is assessed by calculating the ratio between the largest and smallest group variances, alongside performing Levene’s test [39]. Additionally, marginal normality is inspected visually using histograms and Q–Q plots of centralized scores [40]. These diagnostics inform the decision to proceed with either a parametric or a rank-based statistical test.

*Parametric test with repeated-measures ANOVA and Tukey’s HSD*: If parametric assumptions are satisfied, a repeated-measures ANOVA [41] is applied to the matrix of scores $\{m_{i,r}(h)\}$, with the contender model *h* as the within-subjects factor and the resampling split (i, r) as the subject. The global null hypothesis that all contenders have equal mean performance, $H_0: \mu _1 = \mu _2 = \dots = \mu _{|\mathcal {H}|}$, is evaluated using the F-statistic. If $H_0$ is rejected, pairwise post-hoc comparisons are performed with Tukey’s Honest Significant Difference (HSD) test [42], which employs the studentized-range distribution to derive simultaneous confidence intervals for mean differences and adjusted *p*-values that control the family-wise error rate [43]. Results are visualized as multiple-comparisons similarity (MCSim) heatmaps and mean-difference confidence-interval (CI) plots, which together display pairwise significance, effect sizes, and the relative ranking of methods.

*Nonparametric test with Friedman and Conover*: If parametric assumptions fail, the Friedman test [44] is applied to the within-block ranks of the scores, $R_{i,r}(q)$. A significant Friedman statistic is followed by Conover post-hoc pairwise comparisons with Holm multiplicity correction [45, 46], yielding adjusted $p$-values and a pairwise significance matrix. Average ranks $\bar{R}_q$ are computed as the mean of per-fold percentile ranks and used to summarize relative ordering; results are presented graphically as significance heatmaps (sign plots) and critical-difference diagrams (CDD) to convey both pairwise differences and the overall ranking among methods [47].

**Fig. 5:**
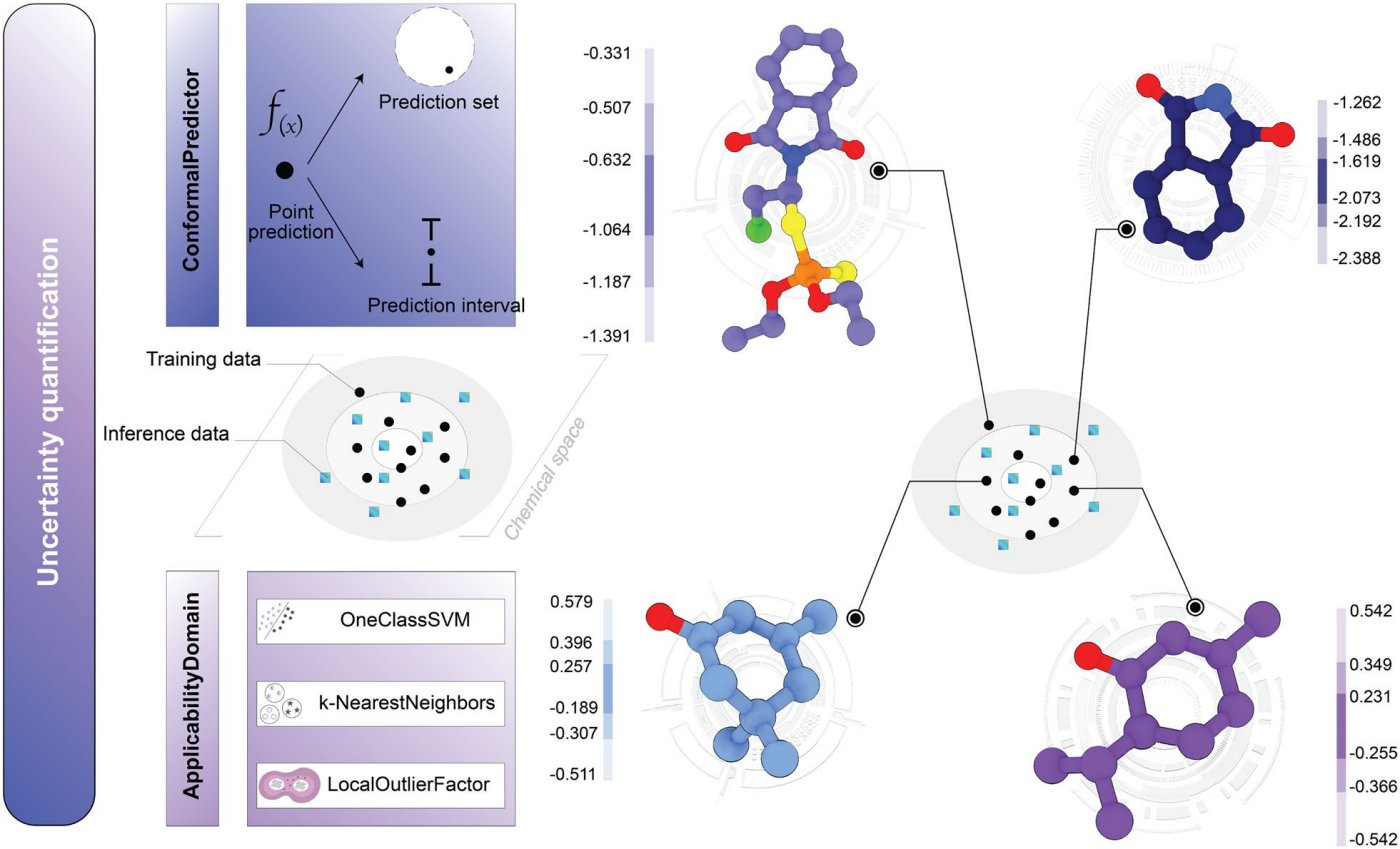
Out-of-distribution detection and uncertainty quantification in ProQSAR: (i) conformal predictors (prediction intervals/sets with calibrated coverage) and (ii) applicability-domain detectors (LOF, *k*-NN density, one-class SVM) to flag out-of-sample compounds

*Out-of-sample detection*: The Evaluation module also implements two complementary (see Fig. 5), training-only procedures for identifying unreliable or out-of-distribution predictions: (i) classical QSAR applicability-domain (AD) estimators and (ii) distribution-free conformal prediction (CP). AD is provided by three detectors [48] fitted on the training features: one-class SVM (RBF, $\gamma$ selectable via auto heuristics) [49], $k$-NN density (mean distance to the $k$ nearest neighbors) [50] and Local Outlier Factor in novelty mode [33]. Each AD method returns a continuous novelty score $a(x)$ which is thresholded (by default at the $100\times \texttt {rate\_of\_outliers}$ percentile) to yield per-sample in/out flags. Key parameters such as the distance metric, $k$, and the thresholding policy are configurable. CP is implemented via MAPIE [51] wrappers, using cross-conformal (CV+) calibration [52] to produce $(1-\alpha )$ marginal prediction intervals (regression) or prediction sets (classification). CP returns per-sample point predictions and, for each requested $a$, the corresponding intervals or sets. In practice AD and CP are produced per sample and can be combined operationally: AD delimits the region of reliable interpolation while CP quantifies calibrated predictive uncertainty, together enabling principled downstream filtering and decision-making.

### Automated pipeline and report

**Fig. 6:**
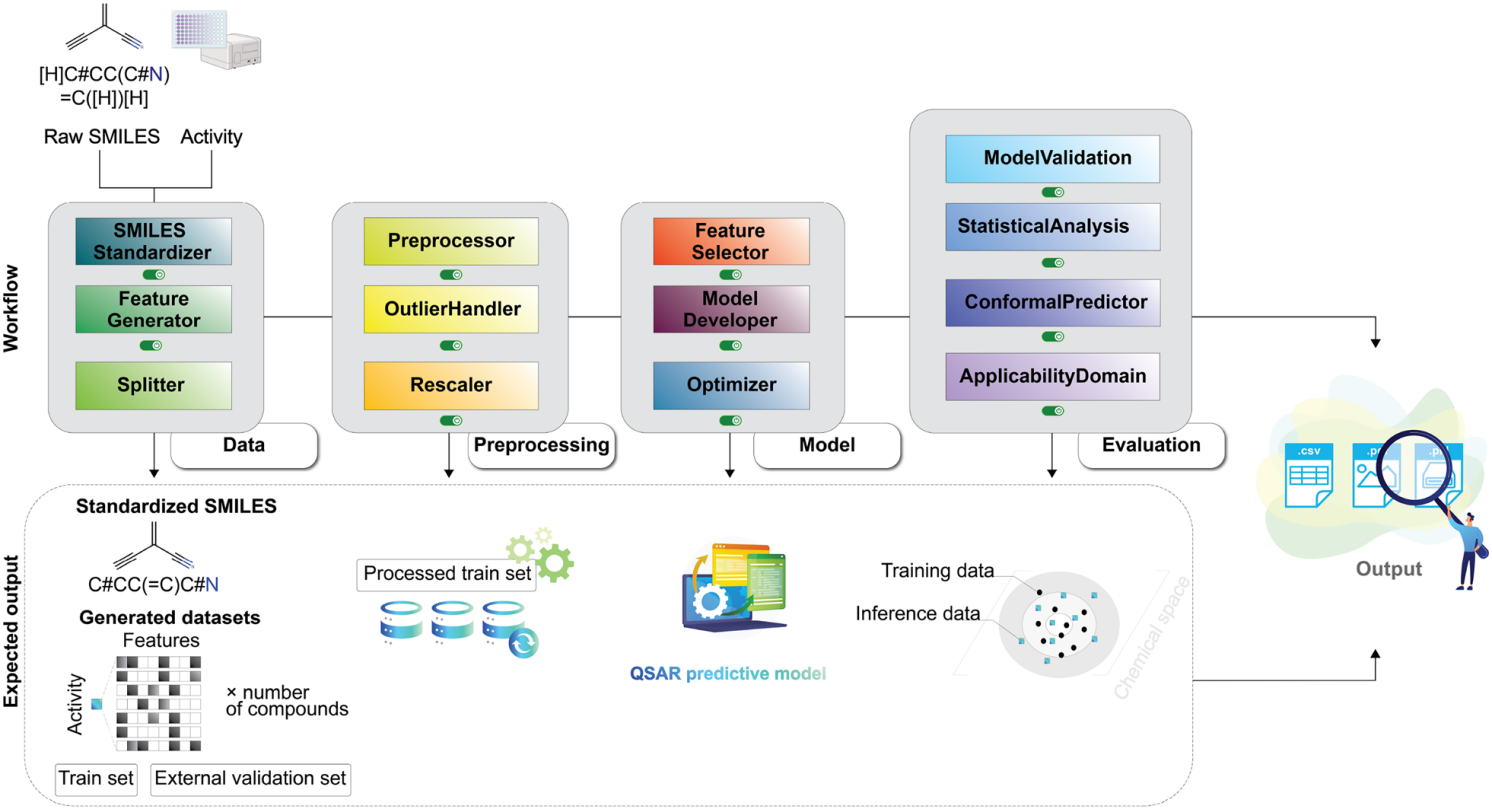
Automated pipeline: descriptor-first evaluation followed by targeted optimization and packaging; all steps are executed with preserved provenance and shared split family $\mathcal {S}$

ProQSAR implements a single-entry automated workflow (see Fig. 6) that executes a user-configurable sequence of modules on a dataset $\mathcal {D}=\{(s_j,y_j)\}_{j=1}^N$. The pipeline enforces a common split family

$$\mathcal {S}=\{(\mathcal {T}_{i,r},\mathcal {V}_{i,r})\}_{i=1\ldots k,\; r=1\ldots R}$$

(for the default configuration $k=5, R=5$, i.e. $5\times 5$ repeated CV) which is reused across feature-selection and model benchmarking stages to permit valid paired statistical comparisons.

*Descriptor-first evaluation*: Let $\Psi =\{\psi _p\}_{p=1}^P$ denote the set of descriptor/fingerprint types (e.g. ECFP4, MACCS, physicochemical suites). The pipeline proceeds as follows:

1. For each $\psi _p\in \Psi$, compute the feature matrix $X^{(\psi _p)}$ and persist it as a separate CSV artifact.
2. Independently for each $X^{(\psi _p)}$ run the sequence: Preprocess $\rightarrow$ FeatureSelector $\rightarrow$ ModelDeveloper, yielding a collection of per-split performance scores
  $$M^{(\psi _p)} = \big \{\, m_{i,r}^{(\psi _p)} \;:\; i=1\ldots k,\; r=1\ldots R \,\big \},$$
  where $m_{i,r}^{(\psi _p)}$ denotes the metric (e.g. $R^2$, F1-score) on the split corresponding to fold $i$ of repeat $r$.
3. Aggregate $\{M^{(\psi _p)}\}$ into a cross-validation report and select $\psi ^\star$ by maximizing the target score’s mean across repeats and folds. Optionally, invoke the Evaluation module for repeated-measures statistical comparisons across descriptor families (e.g., Friedman with post-hoc Conover) before finalizing $\psi ^\star$. The Optimizer is omitted in this stage to reduce computational cost.

*Model optimization and finalization*: Having selected the optimal descriptor $\psi ^\star$, ProQSAR proceeds to hyperparameter search and final fitting:

1. Optionally apply a pre-optimization gate: perform small-budget tuning to screen candidates for feature selection and model development (Sect. “Model”).
2. Run full hyperparameter optimization using Optimizer.
3. Refit the selected estimator on the training data then freeze all transformers and configuration settings.

***Packaging, report, and inference***

- Serialized bundle: Archive with trained transformers, estimator, AD detector, conformal predictor, and split indices for full reproducibility.
- Deployment: Apply the serialized pipeline on raw inputs: identical featurization, frozen preprocessing and feature-selection transforms, and prediction with the fitted estimator. Optionally include conformal intervals/sets and AD flags.
- Comprehensive report: Machine- and human-readable outputs: logs, processed datasets, cross-validation and external validation reports, statistical test outputs with visualizations, and prediction tables.
- Inference: A Python Inference wrapper reproduces the training pipeline deterministically: frozen transformers, fixed seeds, and features $\psi ^\star$.

*Parallel and distributed execution*: ProQSAR utilizes multi-core processing for independent tasks such as molecular featurization and cross-validation folds. The codebase is adaptable for distributed execution on backends such as Dask [53] or Spark [54] to facilitate the analysis of massive chemical libraries.

*Deployment requirements*: ProQSAR operates on Python 3.11 and is distributed via PyPI, Conda, and Docker (see Supporting Section S1).^1^ Standard execution requires a tabular dataset containing SMILES strings and bioactivity values alongside a configuration file to manage the modeling pipeline. Trained models are exported as serialized bundles for automated inference on novel chemical structures via the command line interface or Python API.

## Results

### Benchmarking on MoleculeNet datasets

We evaluate representative MoleculeNet tasks [55] under a unified protocol with standardized preprocessing and Bemis–Murcko scaffold partitioning. Our aim is methodological comparability: we reproduce strong baselines under identical data handling, rather than proposing novel architectures. All models use the same folds, feature visibility, and target transformations; hyperparameters are tuned via cross-validation with fixed RNG (default 42 for all tasks), and we report point estimates with dispersion to reflect stability.

**Table 1:** Selected benchmarks grouped by domain

Domain	Dataset	#Targets	#Samples	Metric
Physical Chemistry	ESOL [56]	1	1128	RMSE
	FreeSolv [57]	1	642	RMSE
	Lipophilicity [58]	1	4200	RMSE
Quantum Mechanics	QM7 [59]	1	6834	MAE
	QM8 [60]	12	21786	MAE
Biophysics / Physiology	BACE [61]	1	1513	ROC–AUC
	BBBP [62]	1	2053	ROC–AUC
	Tox21 [63]	12	7831	ROC–AUC
	SIDER [64]	27	1427	ROC–AUC
	ClinTox [65]	2	1484	ROC–AUC
	Estrogen [66]	2	3122	ROC–AUC
	MetStab [67]	2	2267	ROC–AUC
Activity Cliffs	CHEMBL2835_Ki [68]	1	615	RMSE
	CHEMBL4616_EC50 [68]	1	682	RMSE

Classical ML pipelines excel in low-data regimes but scale less favorably in data/compute than modern deep learning. To mirror wet-lab constraints, we focus on single- or dual-target tasks with moderate sample sizes (Table 1), where reproducibility, calibrated uncertainty, and AD control are often decisive. Furthermore, we extended our evaluation to multi-target benchmarks, including QM8, Tox21, and SIDER, to assess the generalizability of ProQSAR across diverse domains.

#### Regression task

Table 2 compares ProQSAR to state-of-the-art baselines on ESOL, FreeSolv, Lipophilicity (Lipo), and two quantum mechanics datasets (QM7, QM8). ProQSAR attains the best overall score (0.658 ± 0.11), ranking first or second on all datasets in physical chemistry domain, indicating both accuracy and cross-task robustness (low average dispersion).

On ESOL ($n=1128$) and FreeSolv ($n=642$), ProQSAR provides clear gains over self-supervised (MolCLR, MGSSL) and advanced graph models (HiMol, KGG). The improvement on FreeSolv (0.494 vs. 0.731 RMSE for KGG) is substantial and consistent with the pipeline’s emphasis on deterministic preprocessing (tautomer/charge handling, stereochemistry), standardized featurization, and leakage-averse scaling. These components reduce variance and attenuate overfitting in noisy, limited-label settings. On Lipo ($n=4200$), ProQSAR remains competitive (0.706 RMSE) but trails KGG (0.665), reflecting the growing advantage of representation learning as chemical diversity and sample size increase.

**Table 2:** RMSE ($\downarrow$) on physical-chemistry regression benchmarks from MoleculeNet (ESOL, FreeSolv, Lipo) and MAE ($\downarrow$) on quantum-mechanical property prediction datasets (QM7, QM8)

	PhysChem (RMSE $\downarrow$)				Quantum (MAE $\downarrow$)		
Model	ESOL	FreeSolv	Lipo	Avg.	QM7	QM8	Avg.
MolCLR [69]	1.333	3.285	0.720	1.779	104.184	**0**.**0187**	52.101
G-Motif [70]	1.286	4.432	0.779	2.166	222.957	0.0203	111.489
MGSSL [71]	1.346	2.980	0.751	1.692	155.913	0.0198	77.966
HiMol [72]	0.833	2.283	0.708	1.275	91.501	0.0199	45.760
KGG [73]	0.944	0.731	**0**.**665**	0.780	77.6838	0.0190	38.851
ProQSAR (ours)	**0**.**774**	**0**.**494**	0.706	**0**.**658**	**74**.**0874**	0.0225	**37**.**055**

Best results are in **bold**, second-best are underlined

In the quantum mechanics domain, ProQSAR outperformed other methods on the single-task QM7 dataset with an MAE of 74.0874, while remaining competitive on the larger, multi-task QM8 dataset with an MAE of 0.0225. This performance gap may be attributed to the architectural design of ProQSAR as a single-task learning framework, whereas modern deep learning models leverage multi-task learning to enhance generalization. Furthermore, we extended our evaluation to activity cliff datasets from MoleculeACE [68], specifically CHEMBL2835_Ki and CHEMBL4616_EC50, yielding RMSE values of 0.5338 and 0.8048, respectively.

#### Classification task

For classification tasks, we employ the area under the receiver operating characteristic curve (ROC–AUC) as the primary metric to evaluate predictive performance and model discriminative power. Table 3 summarizes shared classification endpoints (BACE, BBBP, Tox21, SIDER, ClinTox) and two auxiliary endpoints (Estrogen, MetStab). The average performance reflects only the shared datasets to ensure direct comparability. ProQSAR is strongest on ClinTox (91.4 ROC–AUC, best overall) but underperforms on BACE (71.4) and BBBP (63.7), yielding an average of 70.4 ± 11.6. On the remaining shared endpoints, ProQSAR is moderate on Tox21 (68.7) and SIDER (56.9) (Table 3). This aligns well with our observations in the quantum mechanics domain, where ProQSAR performs competitively on single-task learning but underperforms in multi-task scenarios. Consequently, ProQSAR remains competitive in single-task scenarios but tends to underperform on multi-target datasets, such as Tox21 (12 targets) and SIDER (27 targets). We also report auxiliary results on Estrogen (93.0) and MetStab (68.4) without including them in the shared-dataset average. Dataset scale and class structure contextualize this pattern: on smaller, noisier tasks such as ClinTox ($n=1484$; two targets), where stringent preprocessing and leakage-averse scaling are beneficial; on BACE ($n=1513$) and BBBP ($n=2053$), representation-learning methods (HiMol, KGG) gain from richer, task-tuned embeddings.

**Table 3:** ROC–AUC ($\uparrow$) on MoleculeNet classification benchmarks

Model	BACE	BBBP	Tox21	SIDER	ClinTox	MetStab	Estrogen	Avg.
MolCLR	76.5	69.3	74.2	56.4	90.4	—	—	73.4
G-Motif	81.1	68.6	73.3	59.8	78.9	—	—	72.3
MGSSL	79.1	69.7	76.5	61.8	80.7	—	—	73.6
HiMol	84.3	73.2	76.2	61.3	80.8	—	—	75.2
KGG	**86**.**3**	72.5	75.6	**64**.**9**	87.3	—	—	77.3
ProQSAR (ours)	71.4	63.7	68.7	56.9	**91**.**4**	68.4	93.0	70.4

Best values are in **bold**, second-best are underlined. Avg. is computed over the five shared datasets only

The higher dispersion (i) for ProQSAR (11.6) indicates sensitivity to dataset characteristics (class imbalance, label noise). (ii) For BACE/BBBP, gains of HiMol/KGG imply that task-specific graph encoders capture non-linear structure–activity relationships beyond fixed descriptors. (iii) Complementarity emerges: ProQSAR is attractive when labels are scarce and auditability matters; deep models dominate as data scale and homogeneity increase.

### Case study on FreeSolv dataset

As an illustrative example, we examine the FreeSolv dataset, a standard MoleculeNet benchmark comprising 642 small organic molecules with experimentally measured hydration free energies (kcal/mol) [55]. Hydration free energy is a key physicochemical property governing solubility, permeability, and bioavailability, and thus serves as a stringent testbed for QSAR validation.

**Table 4:** OECD validation principles applied to the FreeSolv dataset

Principle	Implementation in ProQSAR	Support
Defined endpoint	Hydration free energy, experimentally measured; continuous and interpretable property	✓
Unambiguous algorithm	Deterministic preprocessing, fixed feature sets, controlled random seeds, defined algorithms	✓
Applicability domain	LOF detector; flags out-of-scope molecules	✓
Goodness-of-fit, robustness, and predictivity	$k \times R$ cross-validation, scaffold-aware splits	✓
Mechanistic interpretation	Feature attribution under development	✗

Model development was aligned with the OECD principles for QSAR validation [74]. Table 4 summarizes compliance in the context of ProQSAR.

#### Optimal dataset selection

In the default configuration, we evaluated 14 distinct descriptor and fingerprint types: ECFP2, ECFP4, ECFP6, FCFP2, FCFP4, FCFP6, MACCS, RDK5, RDK6, RDK7, avalon, mordred, pubchem, and rdkdes (RDKit descriptors). The entire process is automated through the proqsar.optimal_dataset routine described in Sect. “Automated pipeline and report”.

**Fig. 7:**
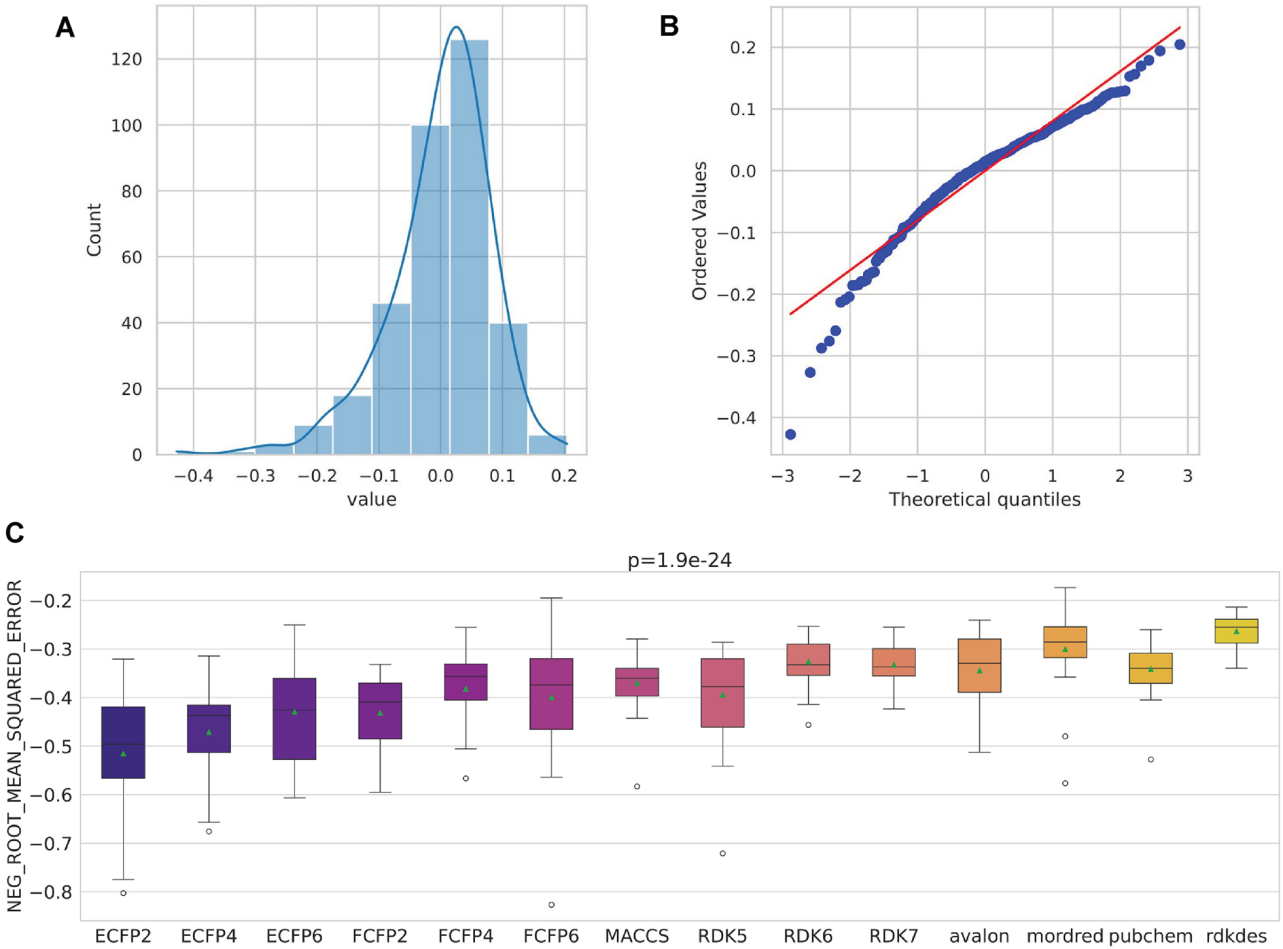
Assessment of score distributions for descriptor/fingerprint sets. (A) Histogram of cross-validated scores. (B) Q–Q plot versus Gaussian distribution. (C) Friedman repeated-measures test results

Model assessment was conducted using $5 \times 5$ cross-validation, yielding independent error estimates for each representation. The resulting score distributions were subjected to a normality test (see Fig. 7A–B). The histogram and Q–Q plot suggest approximate *Gaussianity*. Next, we evaluated homogeneity of variance using Levene’s test. The test yielded an F-statistic of approximately 14.33 with an associated *p*-value $< 0.05$, leading to rejection of the null hypothesis of equal variances. This indicates significant heteroscedasticity among descriptor sets, necessitating the use of non-parametric methods. Consequently, we applied the non-parametric Friedman repeated-measures test (Fig. 7C). It returned a *p*-value $< 0.05$, indicating that at least one descriptor set performs significantly differently from the others.

**Fig. 8:**
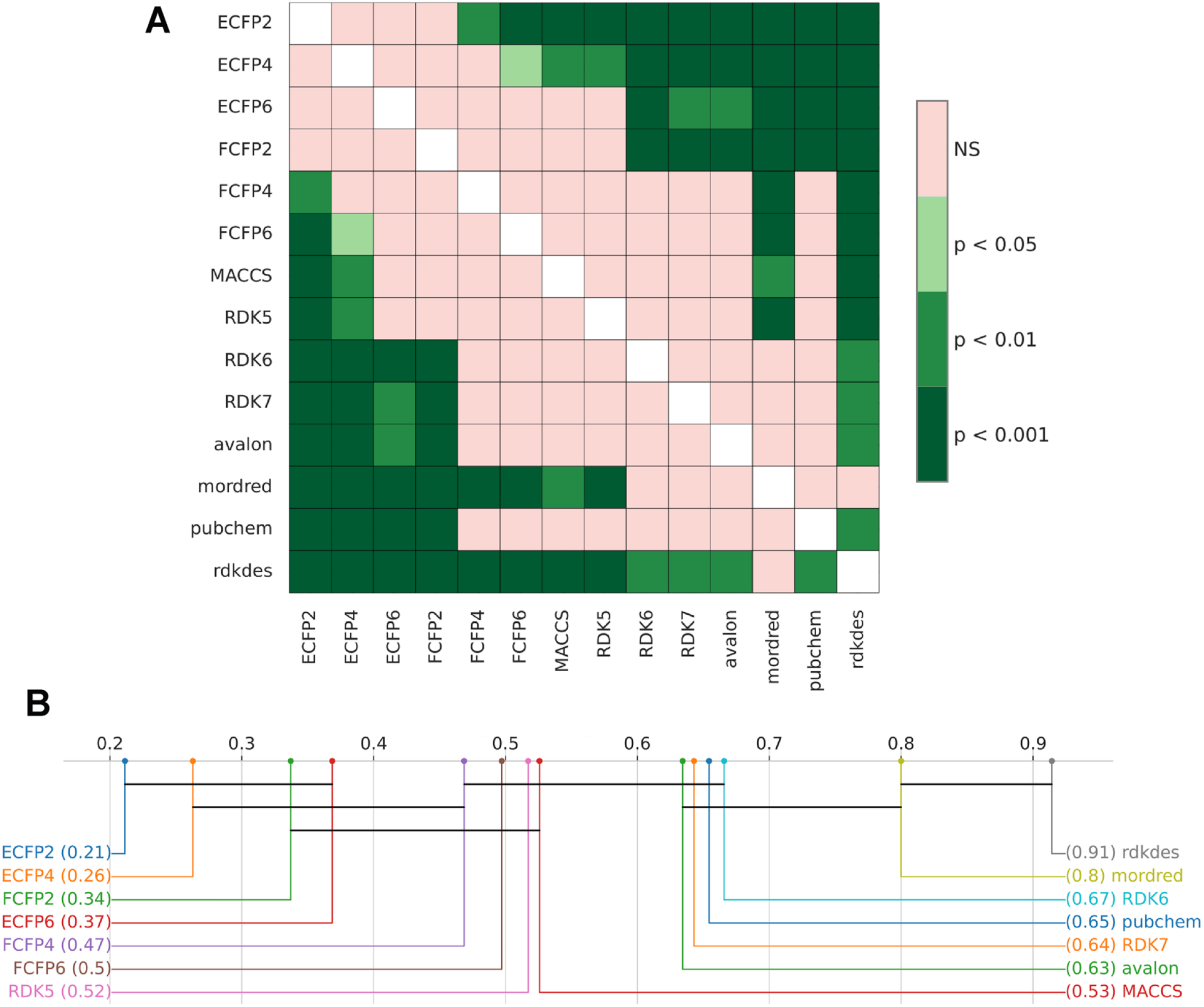
Post-hoc statistical analysis of descriptor/fingerprint performance. (A) Significance plot of pairwise Conover–Friedman tests. (B) Critical difference diagram

Post-hoc comparisons were then conducted using the Conover–Friedman test (see Fig. 8). As shown in Fig. 8B, rdkdes achieved optimal average negative root mean squared error ($-0.264$) (see Table S4) and was significantly different from most alternatives, with the exception of mordred. Although mordred includes a substantially larger set of features, its performance was not statistically superior. Therefore, given both statistical evidence and computational efficiency, rdkdes was selected as the optimal representation for downstream training and optimization.

#### Model development and optimization

Model development and hyperparameter optimization followed the same reproducible protocol used for representation selection. For each candidate feature selector and learning algorithm we obtained independent performance estimates via $5\times 5$ repeated cross-validation; summary statistics are reported in Table S5.

**Fig. 9:**
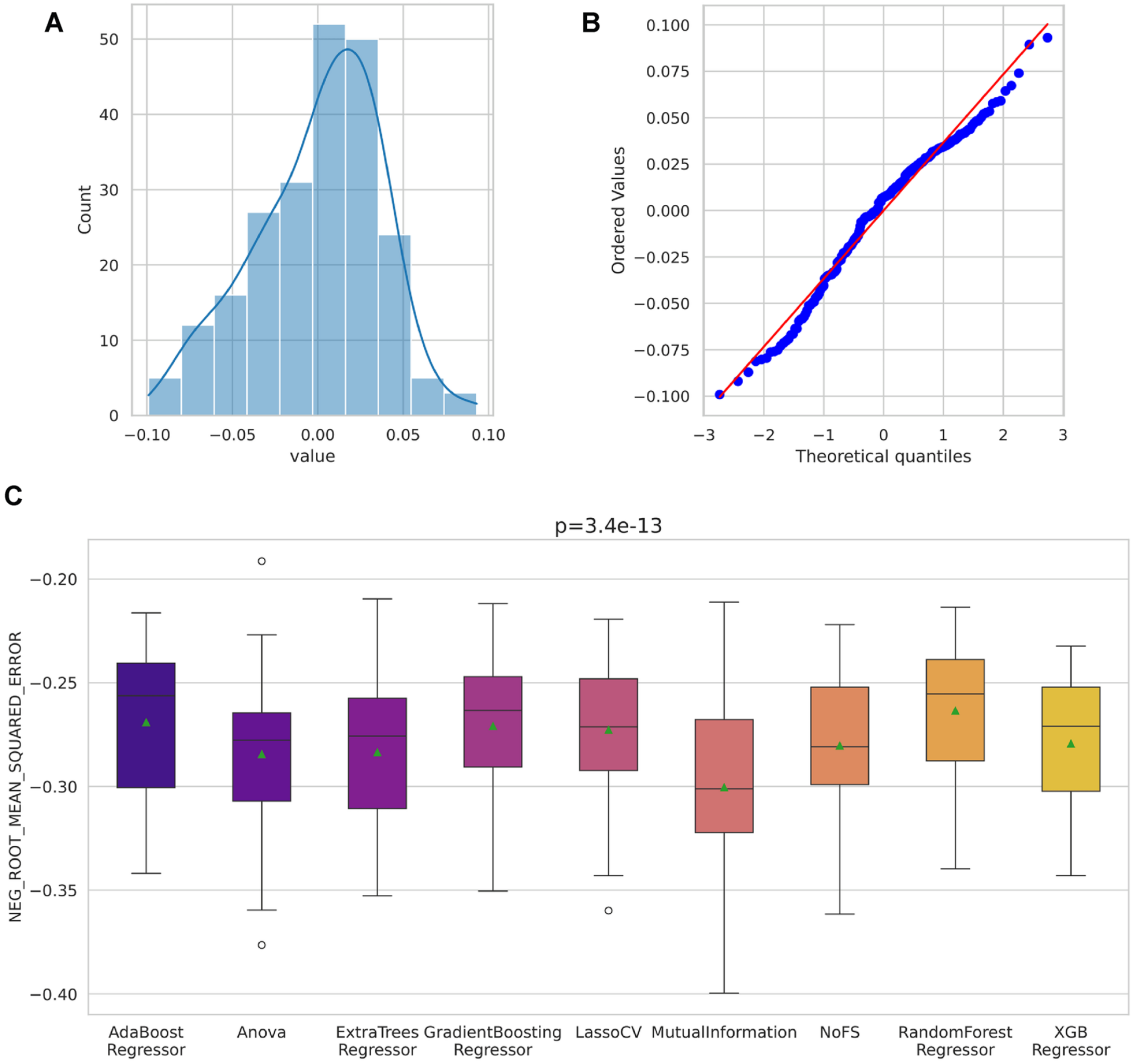
Score distribution diagnostics for feature selection. **A** Histogram of cross-validated RMSEs. **B** Q–Q plot against the normal distribution. **C** Repeated-measures ANOVA results

Prior to formal comparison we examined the score distributions for normality and homoscedasticity. Normality was assessed visually (see Fig. 9A–B), and homogeneity of variance was evaluated with Levene’s test, which returned $F = 2.02$ and $p = 0.79$, indicating no significant variance heterogeneity. Given approximate normality and homoscedasticity, we proceeded with a one-way repeated-measures ANOVA to compare feature selectors. The ANOVA indicated a significant effect of the selector on performance ($p < 0.05$), warranting post-hoc pairwise comparisons.

**Fig. 10:**
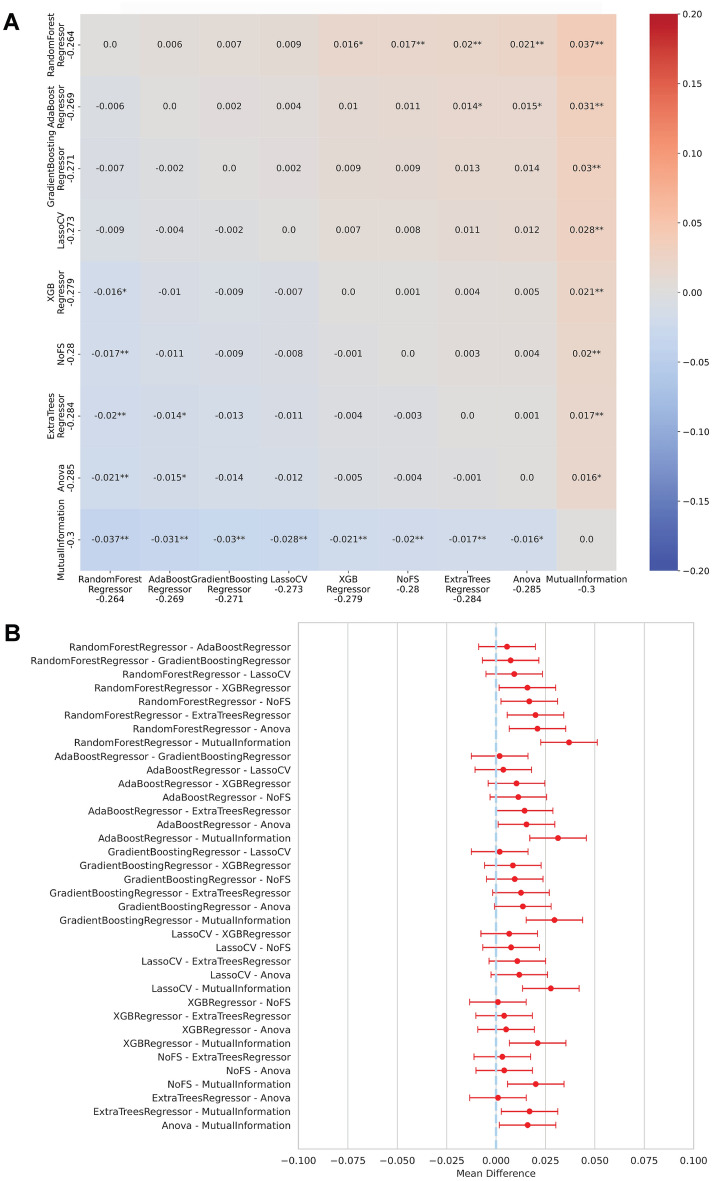
Post-hoc analysis of feature selectors. (A) Tukey’s HSD multiple-comparisons similarity plot. (B) Tukey’s HSD confidence intervals for pairwise differences

Post-hoc pairwise testing was performed using Tukey’s honestly significant difference procedure; results are visualized in Fig. 10. RandomForestRegressor attained the best mean score ($0.2636 \pm 0.0322$, Table S5) and was not significantly different from GradientBoostingRegressor, LassoCV and AdaBoostRegressor in the Tukey comparisons. Because RandomForestRegressor combined top-ranked predictive performance with computational efficiency, it was selected as the preferred feature-selection workflow for downstream optimization.

For model (algorithm) selection we applied the same hypothesis-testing cascade as for representation and feature selection (normality $\rightarrow$ homoscedasticity $\rightarrow$ choice of test). In contrast to the feature-selector stage, model comparisons exhibited significant variance heterogeneity (Levene’s test: $F = 5.88$, $p = 0.0006$); accordingly, we employed a Friedman repeated-measures test followed by Conover post-hoc pairwise comparisons with appropriate multiplicity correction. Because the testing workflow is identical to that described in Sect. “Optimal dataset selection”, we present only the summary comparison in Table S6. Based on cross-validated ranks and post-hoc significance, CatBoostRegressor was selected as the preferred algorithm (mean RMSE $0.2344 \pm 0.0353$). Given the already low error (mean RMSE $0.2344 \pm 0.0353$), extensive hyperparameter exploration is unlikely to produce substantial improvements; accordingly, the CatBoostRegressor was adopted as the base model for external benchmarking and downstream analyses.

### External validation and baseline comparison

**Fig. 11:**
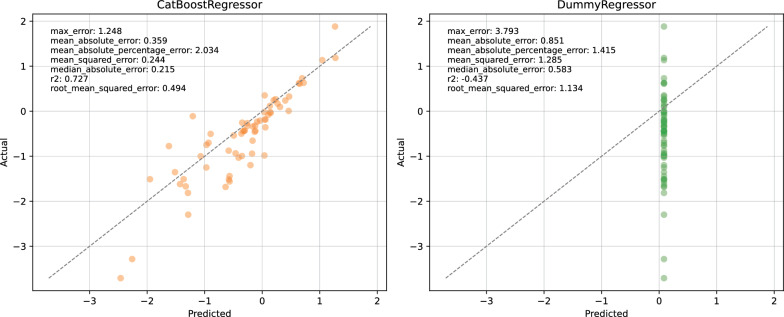
External validation of FreeSolv dataset using CatBoostRegressor comparing against baseline DummyRegressor

External validation on the held-out test set (Fig. 11; Table S7) shows that two learners generalize best: MLPRegressor and CatBoostRegressor, which produce identical summary statistics on this split ($R^{2}=0.73$, $\mathrm{RMSE}=0.49$, $\mathrm{MAE}=0.36$). Although point estimates on the hold-out set are indistinguishable, the cross-validation ranking and subsequent Conover post-hoc comparisons following the Friedman test indicate that CatBoostRegressor is *significantly* superior to MLPRegressor, thereby reinforcing our selection of CatBoostRegressor for the final inference pipeline.

For reference and to establish a minimal performance baseline, a DummyRegressor that predicts the training mean attains substantially poorer performance ($R^{2}=-0.44$, $\mathrm{RMSE}=1.13$, $\mathrm{MAE}=0.85$). Models failing to outperform this constant-value baseline do not capture systematic signal and should not be considered externally valid. Consequently, we report the dummy baseline alongside all candidate models to prevent over-interpretation of marginal improvements.

## Conclusion

ProQSAR is a prescriptive yet customizable, end-to-end QSAR workbench. While it offers a validated default workflow tailored for small-to-moderate hit-to-lead datasets, it also allows users to manually adjust configurations as needed. It encapsulates molecular standardization (tautomer and protonation handling), leakage-safe preprocessing, and configurable descriptor generation (e.g., cLogP, TPSA, ECFP4) behind a compact API and a deployable Inference wrapper so practitioners without deep computer science expertise can reproduce, run and interpret models reliably. The workbench exposes explicit uncertainty and applicability-domain diagnostics together with repeated $k\times R$ cross-validation, producing well-calibrated potency predictions. Empirically, ProQSAR offers a robust, easy-to-run baseline across the MoleculeNet suite. For regression tasks, our results demonstrate that ProQSAR excels on smaller-scale, single-task benchmarks such as ESOL, FreeSolv, and QM7, while maintaining competitive performance on multi-task datasets like QM8. A similar trend is observed in classification, where ProQSAR achieves superior results on the ClinTox dataset. Taken together, these findings underscore ProQSAR’s utility as a robust baseline for methodological benchmarking and a reliable tool for rapid deployment in resource-constrained or applied settings.

Despite these strengths, ProQSAR makes explicit design trade-offs: prioritizing reproducibility and interpretability increases resampling and descriptor I/O overhead for very large or streaming datasets, and newly labeled data still require full retraining. We are addressing these limits by adding adaptive caching and lazy descriptor computation, distributed execution (Dask/Spark) with stratified subsampling and surrogate-based tuning, incremental partial-fit wrappers and automated drift detection, and usability features such as GUI presets and per-split attribution summaries. Together, these enhancements aim to accelerate DMTA cycles while preserving ProQSAR’s transparency, traceability, and ease of adoption.

## Additional file

Supplementary file 1 (pdf 869 KB)

## Data Availability

The datasets supporting the conclusions of this article are available in the GitHub repository: https://github.com/Medicine-Artificial-Intelligence/ProQSAR. The source code is available at the same repository (https://github.com/Medicine-Artificial-Intelligence/ProQSAR) and has been archived on Zenodo: https://zenodo.org/records/17209616.

## Footnotes

1 Documentation: https://proqsar.readthedocs.io/

## Declarations

### Availability and requirements

Lists the following:

- Project name: e.g. My bioinformatics project
- Project home page: e.g. http://sourceforge.net/projects/mged
- Operating system(s): e.g. Platform independent
- Programming language: e.g. Java
- Other requirements: e.g. Java 1.3.1 or higher, Tomcat 4.0 or higher
- License: e.g. GNU GPL, FreeBSD etc. Any restrictions to use by non-academics: e.g. licence needed
