# Supplementary material for: ProQSAR: A modular and reproducible framework for small-data QSAR modeling with fit-and-use models
Source: J Cheminform. 2026 Apr 22;18:52. doi: 10.1186/s13321-026-01175-9 (PMC13123191; doi:10.1186/s13321-026-01175-9)
Supplement: Supplementary file 1 — (pdf 869 KB) [file 13321_2026_1175_MOESM1_ESM.pdf]

Supporting Information

S1 Software Architecture and installation

`ProQSAR` is an opinionated, end-to-end Quantitative Structure-Activity Relationship (QSAR) toolkit built for reproducible model development and production-ready inference. The package organizes the typical QSAR workflow into a small set of focused modules that together support dataset standardization, featurization, preprocessing, model benchmarking, hyperparameter optimization, applicability domain and conformal prediction, and a compact inference API for deployment, illustrated in Figure S1.

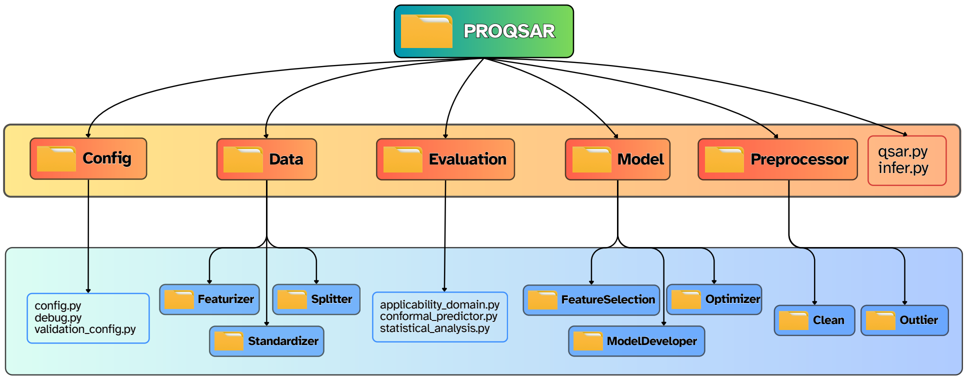

Fig. S1: High-level architecture of `ProQSAR`.

Installation

Binary packages and container images are available for convenience.

Listing 1: Install via PyPI

```
pip install --upgrade proqsar
```

Listing 2: Install via conda (user channel)

```
conda install -c tieulongphan
```

Listing 3: Pull Docker image

```
docker pull tieulongphan/proqsar:latest
```

Dependencies

The toolkit builds on widely adopted scientific `Python` libraries. For reproducible results, it is strongly recommended pinning versions in the environment. The table below lists commonly used packages and the versions used in our reference release.

Table S1: Representative dependency versions for the `ProQSAR` reference release.

Package	Version	Package	Version
`rdkit` [20]	2025.03.1	`seaborn` [75]	0.13.2
`pandas` [76]	2.2.2	`joblib` [77]	1.4.2
`scikit-learn` [78]	1.5.1	`xgboost` [79]	2.1.1
`catboost` [23]	1.2.7	`optuna` [24]	4.0.0
`numpy` [80]	1.26.4	`matplotlib` [81]	3.9.2
`scikit-posthocs` [82]	0.11.2	`pingouin` [83]	0.5.5
`scipy` [84]	1.14.1	`statsmodels` [43]	0.14.4
`mapie` [85]	0.9.2	`mordred` [86]	1.2.0

Documentation

Full examples, the pipeline API and inference documentation are hosted online:

http://proqsar.readthedocs.io/en/latest/

S2 Notation

Data objects

Raw records are denoted

$$\mathcal{D} = \{(s_j, y_j)\}_{j=1}^{N},$$

with inputs s_j and targets y_j. After featurization and metadata assembly we write

$$\mathcal{D} = \{(x_i, y_i, z_i)\}_{i=1}^{N}, \qquad x_i \in \mathbb{R}^P, \quad y_i \in \mathcal{Y}, \quad z_i \in \mathcal{Z},$$

with feature matrix $\boldsymbol{X} \in \mathbb{R}^{N \times P}$, target vector $\boldsymbol{y}$, and row-aligned metadata table $\boldsymbol{Z}$.

Index sets and splits

A deterministic partition defines disjoint index sets

$$\mathcal{I}_{\text{tr}},\ \mathcal{I}_{\text{te}} \subset \{1, \dots, N\}, \qquad \mathcal{I}_{\text{tr}} \cup \mathcal{I}_{\text{te}} = \{1, \dots, N\}.$$

For any $S \in \{\text{tr}, \text{te}\}$ we write $\boldsymbol{X}_S = \boldsymbol{X}[\mathcal{I}_S, :]$ and $\boldsymbol{Z}_S = \boldsymbol{Z}[\mathcal{I}_S, :]$. A family of resampling splits (restricted to the training pool) is

$$\mathcal{S} = \big\{(\mathcal{T}_{i,r}, \mathcal{V}_{i,r})\big\}_{i=1,\ r=1}^{k,R},$$

where i indexes folds and r independent repetitions; $\mathcal{T}_{i,r}, \mathcal{V}_{i,r} \subseteq \mathcal{I}_{\text{tr}}$ with $\mathcal{T}_{i,r} \cap \mathcal{V}_{i,r} = \emptyset$, and for each r, $\bigsqcup_{i=1}^{k} \mathcal{V}_{i,r} = \mathcal{I}_{\text{tr}}$. We denote $n := kR$.

Randomness

$\mathcal{R}(\rho)$ denotes a pseudo-random generator initialized with seed ρ; when repetitions are used we persist $\{\rho_r\}_{r=1}^{R}$.

Metrics and orientation

For a scalar performance metric m, set $s(m) = +1$ if larger is better (e.g. R^2, ROC–AUC, PR–AUC, accuracy) and $s(m) = -1$ if smaller is better (e.g. RMSE, MAE). The oriented metric is $\tilde{m} = s(m)\, m$.

Symbol policy

In this supplementary, we reserve α exclusively for statistical significance/confidence levels (e.g. hypothesis tests, confidence intervals, conformal significance). The dataset test/holdout fraction is denoted p_{test}, the outlier rate is η, RNG seeds for repetitions are ρ_r, and generic thresholds use τ.

S3 Data partitioning

Let the full dataset be $\mathcal{D} = \{(s_j, y_j)\}_{j=1}^{N}$. The `Splitter` generates a single external train/test partition $(\mathcal{D}^{\mathrm{tr}}, \mathcal{D}^{\mathrm{te}})$ based on a chosen strategy. This split is controlled by a test fraction p_{test} (default 0.2) and a single random number generator (RNG) seed ρ (default $\rho = 42$).

The partition is formally defined by disjoint index sets, $\mathcal{I}^{\mathrm{tr}}$ and $\mathcal{I}^{\mathrm{te}}$, such that:

$$\mathcal{D}^{\mathrm{tr}} = \{(s_j, y_j) \colon j \in \mathcal{I}^{\mathrm{tr}}\}, \qquad \mathcal{D}^{\mathrm{te}} = \{(s_j, y_j) \colon j \in \mathcal{I}^{\mathrm{te}}\}$$

where $\mathcal{I}^{\mathrm{tr}} \cup \mathcal{I}^{\mathrm{te}} = \{1, \ldots, N\}$ and $\mathcal{I}^{\mathrm{tr}} \cap \mathcal{I}^{\mathrm{te}} = \varnothing$. The `Splitter` persists the resulting train/test data files and any auxiliary assignments. The available partitioning strategies are:

- **Random holdout:** Uses a repeatable RNG, $\mathcal{R}(\rho)$, to randomly assign indices to the test set until the p_{test} fraction is met.
- **Stratified random holdout:** For classification tasks. Partitions indices into strata based on class labels. Assigns indices randomly within each stratum using $\mathcal{R}(\rho)$ to ensure the test set preserves the original class proportions.
- **Scaffold holdout:** Computes a deterministic Bemis–Murcko scaffold map $\phi_{\mathrm{scaf}} : s_j \mapsto \sigma_j$ (using `rdkit.Chem.Scaffolds.MurckoScaffold`) and forms scaffold groups $G_\ell = \{j : \sigma_j = \sigma_\ell\}$. A deterministic group allocation (Algorithm S1) assigns entire groups, prioritizing the assignment of larger groups to the training set.
- **Random scaffold holdout:** Compute Bemis–Murcko scaffold groups G_ℓ. Use a randomized group allocation (controlled by ρ, Algorithm S2) to assign whole scaffold groups to the test set until p_{test} is reached.
- **Stratified scaffold holdout:** Compute Bemis–Murcko scaffold groups G_ℓ and aggregate each group's activity ("median" or "mean"). Discretize scaffold-level activity into quantile strata and perform a grouped, stratified k-fold partitioning (default $k = 5$). One fold is held out as the test set (test fraction $= 1/k$), with optional shuffling controlled by the RNG seed ρ.
- **Butina cluster holdout.**Computes molecular fingerprints $x_j = f(s_j)$ and their pairwise Tanimoto distances. Run Butina clustering (from `rdkit.ML.Cluster.Butina`) with a cutoff τ (default $\tau = 0.6$) to form clusters C_m. These clusters are then assigned using the same deterministic group allocation routine (Algorithm S1) as the **Scaffold holdout** method.

Algorithm S1 Deterministic group allocation

Require: indices $[N] = \{1, \ldots, N\}$, groups $\mathcal{G} = \{G_i\}$, test fraction $p_{\text{test}} \in (0, 1)$
Ensure: train indices $\mathcal{T}$, test indices $\mathcal{V}$

Let frac_train $\leftarrow 1 - p_{\text{test}}$ and train_cutoff $\leftarrow$ frac_train $\times N$
Sort groups by size (descending) and tie-break by their largest index (descending):

$$\mathcal{G}' \leftarrow \text{sort}\big(\mathcal{G};\ \text{key} = (|G|, \max G),\ \text{reverse}\big)$$

Initialize: $\mathcal{T} \leftarrow \varnothing,\ \mathcal{V} \leftarrow \varnothing$
for each group G in $\mathcal{G}'$ **do**
    if $|\mathcal{T}| + |G| \leq$ train_cutoff **then**
        $\mathcal{T} \leftarrow \mathcal{T} \cup G$ ▷ prioritize placing larger scaffolds in training set
    else
        $\mathcal{V} \leftarrow \mathcal{V} \cup G$
    end if
end for
assert $\mathcal{T} \cap \mathcal{V} = \varnothing$
assert $\mathcal{T} \cup \mathcal{V} = \{1, \ldots, N\}$ ▷ every index belongs to exactly one split
return $\mathcal{T},\ \mathcal{V}$

Algorithm S2 Randomized group allocation

Require: indices $[N] = \{1, \ldots, N\}$, groups $\mathcal{G} = \{G_i\}$, test frac. $p_{\text{test}} \in (0, 1)$, seed ρ
Ensure: train indices $\mathcal{T}$, test indices $\mathcal{V}$

Initialize RNG: $\mathcal{R} \leftarrow \mathcal{R}(\rho)$
Permute groups: $\mathcal{G}' \leftarrow \text{shuffle}(\mathcal{G}; \mathcal{R})$
$\mathcal{T} \leftarrow \varnothing,\ \mathcal{V} \leftarrow \varnothing,\ N_{\text{test}} \leftarrow \lceil p_{\text{test}} N \rceil$
for each group G in $\mathcal{G}'$ **do**
    if $|\mathcal{V}| + |G| \leq N_{\text{test}}$ **then**
        $\mathcal{V} \leftarrow \mathcal{V} \cup G$
    else
        $\mathcal{T} \leftarrow \mathcal{T} \cup G$
    end if
end for
assert $\mathcal{T} \cap \mathcal{V} = \varnothing$
assert $\mathcal{T} \cup \mathcal{V} = \{1, \ldots, N\}$ ▷ every index belongs to exactly one split
return $\mathcal{T},\ \mathcal{V}$

S4 Data cleaning pipeline

Here, we work with a featurized dataset $\mathcal{D} = \{(x_i, y_i, z_i)\}_{i=1}^{N}$ (features x_i, targets y_i, metadata z_i); detailed notation and symbol definitions are given in Section S2. The cleaning pipeline is a composition of stateful transformers

$$\mathcal{P} = T_R \circ T_{R-1} \circ \cdots \circ T_1, \qquad T_r : \ (\mathbb{R}^{n \times P_{r-1}} \times \mathcal{Z}_{r-1}) \to (\mathbb{R}^{n \times P_r} \times \mathcal{Z}_r),$$

each parameterized by $\theta_r \in \Theta_r$. Every T_r satisfies the `scikit-learn` [21] `fit`/`transform` contract via

$$\theta_r = \psi_r\big(\boldsymbol{X}_{\text{tr}}^{(r-1)}, \boldsymbol{Z}_{\text{tr}}^{(r-1)}\big), \qquad (\boldsymbol{X}_S^{(r)}, \boldsymbol{Z}_S^{(r)}) = \phi_r\big((\boldsymbol{X}_S^{(r-1)}, \boldsymbol{Z}_S^{(r-1)}); \theta_r\big),$$

where ψ_r uses only the training indices $\mathcal{I}_{\text{tr}}$, and ϕ_r is deterministic given θ_r. With fixed hyperparameters, split indices, and RNG seeds, $\mathcal{P}$ is a pure (replayable) map. Moreover, any statistic depending on empirical distributions (means, variances, quantiles, covariance, encoders, imputation models, thresholds, etc.) is *fitted on* $\boldsymbol{X}_{\text{tr}}$ *only* and then applied to val/te, which can prevent data leakage.

S4.1 Deduplication

We treat duplicates *at the feature level*: distinct records whose featurized vectors are identical (or numerically indistinguishable), indicating a representation-collision (limited discriminability of the featurizer). Formally, for $x_i, x_j \in \mathbb{R}^P$,

$$i \sim j \iff \|x_i - x_j\| \le \varepsilon,$$

with tolerance $\varepsilon \ge 0$ (take $\varepsilon = 0$ for exact equality; a small ε absorbs floating-point quantization). The equivalence classes $\{C_\ell\}_{\ell=1}^{L}$ induced by $\sim$ are *duplicate groups*; those with $|C_\ell| > 1$ are collapsed to a single representative.

A deterministic `DuplicateHandler` maps each group to one representative:

$$\psi : \ (C_\ell, y_{C_\ell}, z_{C_\ell}) \ \mapsto \ (i_\ell^\star, y_\ell^\star, z_\ell^\star).$$

Supported policies include

$$\begin{aligned}
&\texttt{retain-first/last}, \\
&\texttt{retain-random}: \ i_\ell^\star \sim \text{Unif}(C_\ell) \ \text{(deterministic given seed } \rho), \\
&\texttt{retain-max}: \ i_\ell^\star = \arg\max_{i \in C_\ell} y_i, \qquad \texttt{retain-min}: \ i_\ell^\star = \arg\min_{i \in C_\ell} y_i, \\
&\texttt{aggregate-mean}: \ y_\ell^\star = \tfrac{1}{|C_\ell|} \sum_{i \in C_\ell} y_i, \\
&\texttt{aggregate-median}: \ y_\ell^\star = \text{median}\{y_i\}_{i \in C_\ell}.
\end{aligned}$$

For aggregation policies (`aggregate-mean`/`aggregate-median`) the group's first row is retained as the representative and its activity is replaced by $y_\ell^\star$. We persist the

following metadata: (i) the indices of retained representatives as they appear in the transformed output; (ii) the chosen policy label ψ (and, when `retain-random` is used, the seed ρ for determinism); and (iii) persisted artifacts: identified duplicate columns, the list of dropped original indices, and the exported, transformed dataset.

S4.2 Missing–value remediation

Let the observation mask be $\Omega \in \{0,1\}^{N\times P}$ with $\Omega_{ij} = 1$ if x_{ij} is observed. The feature–wise missing rate is

$$\hat{\pi}_j = 1 - \frac{1}{N}\sum_{i=1}^{N}\Omega_{ij}.$$

The handler performs:

- **Column removal:** Drop feature j if $\hat{\pi}_j > \tau_{\mathrm{drop}}$. In the implementation τ_{drop} corresponds to the `missing_thresh` parameter (interpreted as a percentage; default 40%).
- **Univariate imputation:** For numeric features we support `mean`, `median` or `mode`; categorical and binary columns use the most-frequent category. Concretely, using the training index set $\mathcal{I}_{\mathrm{tr}}$,

$$\widehat{\mu}_{j,\mathrm{tr}} = \frac{\sum_{i\in\mathcal{I}_{\mathrm{tr}}}\Omega_{ij}x_{ij}}{\sum_{i\in\mathcal{I}_{\mathrm{tr}}}\Omega_{ij}}, \qquad \widehat{m}_{j,\mathrm{tr}} = \mathrm{median}\{x_{ij} : i\in\mathcal{I}_{\mathrm{tr}},\ \Omega_{ij}=1\}.$$

- **k-NN imputation:** For a row i and a missing entry at feature j ($\Omega_{ij} = 0$), let

$$J_{it} = \{q : \Omega_{iq}\Omega_{tq} = 1\}$$

  be the set of features observed in both rows i and t. Define the available-features distance

$$d(i,t) = \Big(\frac{1}{|J_{it}|}\sum_{q\in J_{it}}(x_{iq}-x_{tq})^2\Big)^{1/2}.$$

  Denoting by $\mathcal{N}_k(i)$ the k nearest neighbours of i (computed using $d(\cdot,\cdot)$) and using weights w_{it} (uniform by default), the imputed value is

$$\tilde{x}_{ij} = \frac{\sum_{t\in\mathcal{N}_k(i)} w_{it}\, x_{tj}}{\sum_{t\in\mathcal{N}_k(i)} w_{it}},$$

  with k controlled by the `n_neighbors` parameter.
- **Iterative (MICE):** For the set $\mathcal{J}$ of features with missing values, posit conditional models

$$x_{\cdot j} \sim f_j\big(X_{\cdot,\setminus j};\beta_j\big), \qquad j\in\mathcal{J},$$

  and iterate Gibbs–style updates over $j \in \mathcal{J}$ until convergence to obtain $\tilde{X}$.

All statistics and models are fitted on $\mathcal{I}_{\mathrm{tr}}$ only and persisted.

S4.3 Low–variance filtering

For a numerical feature j computed on the training indices $\mathcal{I}_{\text{tr}}$, let

$$\mu_{j,\text{tr}} = \frac{1}{N_{\text{tr}}} \sum_{i \in \mathcal{I}_{\text{tr}}} x_{ij}, \quad s^2_{j,\text{tr}} = \frac{1}{N_{\text{tr}} - 1} \sum_{i \in \mathcal{I}_{\text{tr}}} (x_{ij} - \mu_{j,\text{tr}})^2.$$

Drop feature j if $s^2_{j,\text{tr}} \le \tau_{\text{var}}$. *Rationale:* removes near-constant features that degrade conditioning and add computation without predictive signal.

S4.4 Outlier detection and mitigation

Let a detector be a map $\phi : \{1, \dots, N\} \to \{0, 1\}$ (1 = outlier), with parameters λ estimated on $\mathcal{I}_{\text{tr}}$.

Univariate detector (per feature j)

Let $Q_{j,\text{tr}}(\alpha)$ denote the α-quantile of $\boldsymbol{X}_{\text{tr},\cdot j}$, and define the interquartile range

$$\text{IQR}_{j,\text{tr}} \;=\; Q_{j,\text{tr}}(0.75) - Q_{j,\text{tr}}(0.25).$$

The implementation flags univariate outliers using the IQR fence with multiplier κ (default $\kappa = 1.5$):

$$\phi_{\text{IQR},\kappa}(i, j) \;=\; \mathbb{I}\Big[x_{ij} \notin \big(Q_{j,\text{tr}}(0.25) - \kappa\, \text{IQR}_{j,\text{tr}},\; Q_{j,\text{tr}}(0.75) + \kappa\, \text{IQR}_{j,\text{tr}}\big)\Big].$$

Multivariate detectors

Let μ_{tr} and Σ_{tr} (or robust estimates $\hat{\mu}, \hat{\Sigma}$) be computed from $\boldsymbol{X}_{\text{tr}}$. We consider several multivariate tests:

$$\begin{aligned}
\text{Mahalanobis (Empirical Cov.) [87]} :\quad & d^2_M(x) \;=\; (x - \mu_{\text{tr}})^\top \Sigma^{-1}_{\text{tr}} (x - \mu_{\text{tr}}), \\
& \phi_{M,\alpha}(i) = \mathbb{I}\big[d^2_M(x_i) > \chi^2_{P,\,1-\alpha}\big]; \\
\text{MCD (Robust Cov.) [88]} :\quad & (\hat{\mu}, \hat{\Sigma}) = \text{MCD}(\boldsymbol{X}_{\text{tr}}), \\
& \phi_{\text{MCD},\alpha}(i) = \mathbb{I}\big[d^2_M(x_i; \hat{\mu}, \hat{\Sigma}) > \chi^2_{P,1-\alpha}\big]; \\
\text{Local Outlier Factor [89]} :\quad & \phi_{\text{LOF},\tau}(i) = \mathbb{I}[\text{LOF}_k(i) > \tau]; \\
\text{Isolation Forest [90]} :\quad & \phi_{\text{IF},\tau_s}(i) = \mathbb{I}[s(i) > \tau_s]; \\
\text{One-Class SVM [91]} :\quad & \phi_{\text{SVM},\tau_s}(i) = \mathbb{I}[s(i) < \tau_s].
\end{aligned}$$

Remediation

Let $\mathcal{F} = \{i : \phi(i) = 1\}$ be the set of flagged indices. A deterministic remediation map

$$\psi_{\text{rem}} :\; \mathcal{F} \times (\boldsymbol{X}, \boldsymbol{y}, \boldsymbol{Z}) \;\to\; (\boldsymbol{X}', \boldsymbol{y}', \boldsymbol{Z}', \mathcal{I}_{\text{keep}})$$

implements one of several remediation policies:

- **Removal:** Drop rows $i \in \mathcal{F}$. The retained index set $\mathcal{I}_{\text{keep}} = \{1, \dots, N\} \setminus \mathcal{F}$.
- **Winsorization:** Cap outlying values at feature-wise fences. For each feature j define

$$L_j = Q_{j,\text{tr}}(0.25) - \kappa\,\text{IQR}_{j,\text{tr}}, \qquad U_j = Q_{j,\text{tr}}(0.75) + \kappa\,\text{IQR}_{j,\text{tr}},$$

  and set $x'_{ij} = W_{j,\kappa}(x_{ij})$ with

$$W_{j,\kappa}(x) = \min\{\max(x, L_j),\, U_j\}.$$

- **Imputation:** Mark detected univariate outlier entries as `NaN` and delegate imputation to `MissingHandler` (See Section S4.2).
- **Transformation**: Apply a distributional transform (PowerTransformer or QuantileTransformer to `normal` / `uniform`) to the flagged features.
- **Discretization**: Replace continuous values by bins using `KBinHandler`.

For multivariate outliers the current implementation supports only the removal policy.

S4.5 Scaling

For retained numeric features $\mathcal{J} \subseteq \{1, \dots, P\}$, parameters learned on $\boldsymbol{X}_{\text{tr}}$ define:

$$\text{Min-Max: } \tilde{x}_{ij} = \frac{x_{ij} - \min(\boldsymbol{X}_{\text{tr},\cdot j})}{\max(\boldsymbol{X}_{\text{tr},\cdot j}) - \min(\boldsymbol{X}_{\text{tr},\cdot j})};$$

$$\text{Standard: } \tilde{x}_{ij} = \frac{x_{ij} - \mu_{j,\text{tr}}}{\sigma_{j,\text{tr}}}, \quad \sigma^2_{j,\text{tr}} = \frac{1}{N_{\text{tr}} - 1} \sum_{i \in \mathcal{I}_{\text{tr}}} (x_{ij} - \mu_{j,\text{tr}})^2;$$

$$\text{Robust: } \tilde{x}_{ij} = \frac{x_{ij} - \text{median}(\boldsymbol{X}_{\text{tr},\cdot j})}{\text{IQR}_{j,\text{tr}}};$$

S5 Statistical Test

Evaluation consolidates internal resampling (repeated k-fold cross–validation) and external testing within a *paired, repeated–measures* design.

Design and notation

Fix a family of $k \times R$ data splits

$$\mathcal{S} \;=\; \big\{(\mathcal{T}_{i,r}, \mathcal{V}_{i,r})\big\}_{i=1..k,\; r=1..R}, \qquad n := kR,$$

shared by all contenders $H = \{h_1, \ldots, h_Q\}$. For a scalar metric m (regression: R^2, RMSE, MAE; classification: ROC–AUC, PR–AUC, accuracy) define per–block scores

$$m_{i,r}(h_q) \;=\; m\big(h_q \,\big|\, \mathcal{T}_{i,r}, \mathcal{V}_{i,r}\big), \qquad (i,r) \in \mathcal{S},\;\; q = 1, \ldots, Q,$$

and collect the n-vectors $\mathbf{m}(h_q) = (m_{i,r}(h_q))_{(i,r)\in\mathcal{S}}$. To unify orientation, introduce $s(m) \in \{+1, -1\}$ ($+1$ for metrics to maximize, -1 for those to minimize) and use

$$\tilde{m}_{i,r}(h_q) \;=\; s(m)\, m_{i,r}(h_q).$$

Paired comparisons use identical blocks (i,r) across all $h \in H$, eliminating split-induced variance and increasing statistical power.

Block–wise effects and summaries

Index the blocks by $b \in \{1, \ldots, n\}$ (some bijection maps $(i,r) \mapsto b$). Define block–wise paired differences and their summaries

$$d_b(p,q) \;=\; \tilde{m}_b(h_p) - \tilde{m}_b(h_q), \qquad \bar{m}_q \;=\; \frac{1}{n}\sum_{b=1}^{n} \tilde{m}_b(h_q), \qquad \widehat{\sigma}_q^2 \;=\; \frac{1}{n-1}\sum_{b=1}^{n}\big(\tilde{m}_b(h_q) - \bar{m}_q\big)^2.$$

External testing (single held–out set $\mathcal{D}^{\mathrm{te}}$) reports

$$m^{\mathrm{ext}}(h_q) \;=\; m\big(h_q \,\big|\, \mathcal{D}^{\mathrm{te}}\big).$$

Assumption checks

Before parametric inference we assess: (i) homogeneity of variances across methods for the scores $\{\tilde{m}_b(h_q)\}_b$ via calculating the ratio between the largest and smallest group variances and optionally performing Levene's test [39]; (ii) marginal normality by histograms and Q–Q plots of centralized scores $\tilde{m}_b(h_q) - \bar{m}_q$. These diagnostics inform the decision to proceed with either a parametric or a rank-based statistical test.

Parametric path: repeated–measures ANOVA + Tukey HSD

Under approximate normality and homogeneous variances, fit the one–factor repeated–measures model

$$Y_{bq} \;=\; \mu + \tau_q + s_b + \varepsilon_{bq}, \qquad \sum_q \tau_q = 0, \quad \mathbb{E}[\varepsilon_{bq}] = 0,$$

with "subject" b (CV block) and within–subject factor "method" q. The omnibus null

$$H_0:\; \mu_1 = \cdots = \mu_Q \quad\Longleftrightarrow\quad \tau_1 = \cdots = \tau_Q = 0$$

is tested by the F–statistic

$$F \;=\; \frac{\mathrm{MS}_{\mathrm{method}}}{\mathrm{MS}_{\mathrm{res}}} \quad \text{with df } (Q-1,\, (n-1)(Q-1)).$$

Post–hoc contrasts use Tukey's Honest Significant Difference based on the studentized–range q [42]: for each pair (p, q),

$$\widehat{\Delta}_{pq} \;=\; \bar{m}_p - \bar{m}_q, \qquad \mathrm{SE}_{\mathrm{Tukey}} = \sqrt{\frac{2\,\mathrm{MS}_{\mathrm{res}}}{n}},$$

$$\mathrm{CI}_{1-\alpha}:\quad \widehat{\Delta}_{pq} \;\pm\; \frac{q_{1-\alpha;Q,\;\mathrm{df}=(n-1)(Q-1)}}{\sqrt{2}}\;\mathrm{SE}_{\mathrm{Tukey}},$$

with adjusted p-values derived from the studentized–range distribution. We also report standardized effects

$$\delta_{pq} \;=\; \frac{\widehat{\Delta}_{pq}}{\sqrt{\mathrm{MS}_{\mathrm{res}}}}, \qquad \eta^2_{\mathrm{partial}} \;=\; \frac{\mathrm{SS}_{\mathrm{method}}}{\mathrm{SS}_{\mathrm{method}} + \mathrm{SS}_{\mathrm{res}}}.$$

None-parametric (Rank–based) path: Friedman + Conover

When parametric assumptions fail, rank each block b across methods to obtain R_{bq} (average–rank for ties). The Friedman statistic is

$$\chi^2_F \;=\; \frac{12n}{Q(Q+1)} \sum_{q=1}^{Q} \Big(\bar{R}_q - \tfrac{Q+1}{2}\Big)^2, \qquad \bar{R}_q = \frac{1}{n}\sum_{b=1}^{n} R_{bq},$$

and is evaluated using the χ^2_{Q-1} approximation.

A significant χ^2_F is followed by Conover pairwise post-hoc comparisons with Holm multiplicity correction, producing adjusted p-values and a pairwise significance matrix [45, 46]. Conover's pairwise statistic for methods i and j is the Student-t ratio

$$t_{ij} = \frac{|\bar{R}_i - \bar{R}_j|}{\mathrm{SE}_{ij}}, \qquad \mathrm{SE}_{ij} = \sqrt{\frac{2\,S^2}{n}},$$

with

$$S^2 = \frac{n \sum_{b=1}^{n} \sum_{q=1}^{Q} R_{bq}^2 - \sum_{q=1}^{Q} R_q^2}{(n-1)(Q-1)},$$

where $R_q = \sum_{b=1}^{n} R_{bq}$. Results are presented as significance heatmaps (pairwise adjusted p-values) and as critical–difference diagrams based on the average ranks.

S6 Uncertainty estimation

Let $\mathcal{I}_{\rm tr}$ denote the training index set and let $\widetilde{\boldsymbol{X}}_{\rm tr} \in \mathbb{R}^{N_{\rm tr}\times P}$ be the feature matrix obtained after excluding identifier and target columns. The `Evaluation` module implements two complementary, training-only mechanisms for detecting unreliable or out-of-distribution predictions: *(A) applicability-domain (AD) estimators* and *(B) conformal prediction (CP).*

S6.1 Applicability domain (AD)

An AD method returns a real-valued *novelty score* $a : \mathbb{R}^P \to \mathbb{R}$ (larger $\Rightarrow$ more inlier-like). Given the training scores $\{a(x_i)\}_{i\in\mathcal{I}_{\rm tr}}$, a run-level threshold is set by the quantile rule

$$\tau \;=\; Q_{100\eta}\big(\{a(x_i) : i \in \mathcal{I}_{\rm tr}\}\big), \qquad \eta = \texttt{rate\_of\_outliers} \in (0,1), \tag{S1}$$

and the per-sample AD flag is

$$\text{AD\_flag}(x) = \begin{cases} \texttt{in}, & a(x) > \tau, \\ \texttt{out}, & a(x) \le \tau. \end{cases}$$

The module implements the following detectors (all fitted on $\widetilde{\boldsymbol{X}}_{\rm tr}$ only; hyperparameters such as metric, k, η are configurable):

1. **One-class SVM (RBF):** Use the one-class SVM with RBF kernel $K_\gamma(x,x') = \exp(-\gamma\, d_{\rm se}(x,x')^2)$, where $d_{\rm se}$ is the standardized Euclidean distance using training variances s_j^2. When γ = `"auto"` the implementation searches a grid Γ and selects $\widehat{\gamma} = \arg\max_{\gamma\in\Gamma} \mathrm{Var}(\mathrm{vec}(K_\gamma))$, then sets $a(x) = s_{\rm oc}(x)$ equal to the fitted decision function of the OCSVM [49].
2. **k-NN density surrogate:** For a chosen metric and integer $k \ge 1$, let $\bar{d}_k(x)$ be the mean distance from x to its k nearest neighbours in $\widetilde{\boldsymbol{X}}_{\rm tr}$. Define the stabilized inlier score
$$a(x) = \frac{1}{\bar{d}_k(x) + 1},$$
so that larger local densities map to larger scores (small $\bar{d}_k \Rightarrow$ inlier) [50].
3. **Local Outlier Factor (LOF).** A LOF model trained in novelty mode provides a continuous outlierness measure $s_{\rm lof}(x)$ that we use as $a(x)$ after appropriate sign/offset correction [33].

S6.2 Conformal prediction (CP)

Given a fitted point estimator $\widehat{f}$ (regressor or classifier), cross-conformal prediction (CV$^+$) constructs prediction sets/intervals $\widehat{C}_\alpha(x)$ (or $\widehat{S}_\alpha(x)$ in classification). In regression we pool calibration nonconformity scores computed with cross-validation:

$$\varepsilon_i = |y_i - \widehat{f}_{-k(i)}(x_i)|,$$

where $\widehat{f}_{-k(i)}$ is the model trained without fold $k(i)$. Let $\varepsilon_{(1)} \leq \cdots \leq \varepsilon_{(n_{\mathrm{cal}})}$ be the pooled calibration scores in increasing order and define the empirical $(1-\alpha)$-quantile by the order statistic

$$q_{1-\alpha} = \varepsilon_{\left(\lceil (n_{\mathrm{cal}}+1)(1-\alpha)\rceil\right)}.$$

The $(1-\alpha)$ prediction interval is then

$$\widehat{C}_\alpha(x) = \left[\min_{k=1,\dots,K} \widehat{f}_{-k}(x) - q_{1-\alpha},\ \max_{k=1,\dots,K} \widehat{f}_{-k}(x) + q_{1-\alpha}\right].$$

In classification a suitable nonconformity score $s(x, y)$ (for example, a decreasing transform of class probabilities) is used and the prediction set is

$$\widehat{S}_\alpha(x) = \{y :\ s(x, y) \leq q_{1-\alpha}\}.$$

We implement this cross-conformal (CV$^+$) procedure via the `MAPIE` wrappers [52, 51].

S7 Abbreviations and symbols

Table S2: List of acronyms used in this manuscript and their meanings.

Acronym	Meaning
AD	Applicability Domain (novelty/inlier estimation in feature space).
ANOVA / rm-ANOVA	Repeated-measures analysis of variance (within-block designs).
AUC (ROC/PR)	Area under the ROC / Precision–Recall curve.
CD diagram	Critical-difference diagram (summary of average ranks with significance band).
CP	Conformal prediction (distribution-free calibrated sets/intervals).
CV, CV+	Cross-validation; CV+ denotes cross-validated plus variants for CP.
FWER / FDR	Family-wise error rate / False discovery rate.
HSD	Tukey's Honest Significant Difference (post-hoc).
IQR / MAD	Inter-quartile range / Median absolute deviation.
LOF	Local Outlier Factor.
MAPIE	Model-Agnostic Prediction Interval Estimator (`mapie`).
MCD	Minimum Covariance Determinant (robust covariance estimator).
OCSVM	One-class SVM (novelty detection).
RNG	Random number generator.
RMSE / MAE	Root mean-squared error / Mean absolute error.

Table S3: Mathematical symbols and notation used throughout the manuscript.

Symbol	Definition
$\mathcal{D}$	Dataset; raw $\{(s_j, y_j)\}$, featurized $\{(x_i, y_i, m_i)\}$.
$\boldsymbol{X}$, $\boldsymbol{y}$, $\boldsymbol{Z}$	Feature matrix, target vector, metadata table.
N, P	Number of samples and number of features.
$\mathcal{I}_{\text{tr}}$, $\mathcal{I}_{\text{val}}$, $\mathcal{I}_{\text{te}}$	Train / validation / test index sets.
$\mathcal{S}$	Family of splits $\{(\mathcal{T}_{i,r}, \mathcal{V}_{i,r})\}$.
$\mathcal{R}(\rho)$	RNG seeded with ρ.
ϕ_{scaf}	Bemis–Murcko scaffold mapping.
G_ℓ, C_m	Scaffold and clustering groups.
p_{test}	Test/holdout fraction used in splitting.
η	Rate of outliers used to set AD threshold (see Eq. (S1)).
τ	Generic cutoff/threshold (clustering, outlier, frequency, etc.).
α	Statistical significance level (hypothesis tests, conformal coverage); reserved globally for statistics.
$\mathbb{I}[\cdot]$	Indicator function.
$d_M^2(x)$	Mahalanobis distance squared with training (μ, Σ).
$\chi^2_{\nu,1-\alpha}$	$(1-\alpha)$-quantile of $\chi^2(\nu)$.
$\tilde{m}$	Orientation-aligned metric $\tilde{m} = s(m)\, m$.
$\mathcal{I}_{\text{keep}}$	Indices retained after deduplication/remediation.

S8 Additional Tables and Figures

Table S4: Cross-validated RSME (mean ± std) across 14 descriptor sets.

**Descriptor**	**RMSE** (↓)
ECFP2	0.515 ± 0.122
ECFP4	0.471 ± 0.095
ECFP6	0.429 ± 0.101
FCFP2	0.431 ± 0.076
FCFP4	0.382 ± 0.075
FCFP6	0.399 ± 0.121
MACCS	0.370 ± 0.058
RDK5	0.394 ± 0.099
RDK6	0.326 ± 0.049
RDK7	0.333 ± 0.047
Avalon	0.344 ± 0.082
Mordred	0.300 ± 0.081
PubChem	0.342 ± 0.055
RDKDes	0.264 ± 0.032

Table S5: Cross-validated RSME (mean ± std) for each feature-selection strategy.

**Strategy**	**RMSE** (↓)
AdaBoostRegressor	0.2691 ± 0.0368
Anova	0.2845 ± 0.0394
ExtraTreesRegressor	0.2835 ± 0.0379
GradientBoostingRegressor	0.2709 ± 0.0354
LassoCV	0.2728 ± 0.0346
MutualInformation	0.3005 ± 0.0455
NoFS	0.2804 ± 0.0346
RandomForestRegressor	0.2636 ± 0.0322
XGBRegressor	0.2794 ± 0.0320

Table S6: Cross-validated RSME (mean $\pm$ std) for each machine learning algorithm.

**Model**	**RMSE** (↓)
AdaBoostRegressor	0.3297 ± 0.0303
CatBoostRegressor	0.2344 ± 0.0353
DummyRegressor	0.9045 ± 0.0702
ElasticNetCV	0.3797 ± 0.0379
ExtraTreesRegressor	0.2480 ± 0.0359
GradientBoostingRegressor	0.2425 ± 0.0289
KNeighborsRegressor	0.3240 ± 0.0428
LinearRegression	0.3784 ± 0.0362
MLPRegressor	0.2941 ± 0.0310
RandomForestRegressor	0.2636 ± 0.0322
Ridge	0.3787 ± 0.0372
SVR	0.3146 ± 0.0468
XGBRegressor	0.2635 ± 0.0359

Table S7: External validation metrics on the held-out test set.

**Model**	R^2 (↑)	**RMSE** (↓)	**MAE** (↓)
MLPRegressor	0.7307	0.4907	0.3644
CatBoostRegressor	0.7275	0.4936	0.3590
Ridge	0.6179	0.5845	0.4232
LinearRegression	0.6273	0.5773	0.4158
ElasticNetCV	0.6224	0.5810	0.4200
KNeighborsRegressor	0.5706	0.6197	0.4755
GradientBoostingRegressor	0.5162	0.6577	0.4965
ExtraTreesRegressor	0.5176	0.6567	0.4898
XGBRegressor	0.5088	0.6627	0.4845
SVR	0.6638	0.5483	0.4012
AdaBoostRegressor	0.4942	0.6725	0.5355
RandomForestRegressor	0.4010	0.7318	0.5355
DummyRegressor	-0.4373	1.1336	0.8509
